# Context matters: A meta-ethnography investigating barriers and facilitators for the effective implementation of gambling harm prevention and reduction policies

**DOI:** 10.1371/journal.pone.0343595

**Published:** 2026-02-25

**Authors:** Jani Selin, Lauri Kauppila

**Affiliations:** Department of Healthcare and Social Welfare, Finnish Institute for Health and Welfare, Helsinki, Uusimaa, Finland; Utah State University, UNITED STATES OF AMERICA

## Abstract

This meta-ethnography investigates how contextual factors influence the implementation and effectiveness of gambling harm prevention and reduction interventions. Despite growing recognition of gambling as a public health concern, many interventions focus on individual responsibility and lack evidence of effectiveness. This study synthesizes qualitative research to examine how social, institutional, and cultural contexts shape seven intervention types: educational campaigns, exclusion programs, availability regulations, spending limits, advertising restrictions, behavioral feedback, and behavioral interruptions. A systematic literature search was conducted across multiple databases (Medline, PsycINFO, SocINDEX, Applied Social Sciences Index and Abstracts, Worldwide Political Science Abstracts, Web of Science, and Google Scholar). A quality appraisal using the CASP checklist identified eight methodologically weak or low-quality studies, which were excluded from the synthesis. Fifty-one peer-reviewed qualitative studies were reviewed, with 37 included in the final synthesis. The findings highlight a core tension between harm prevention and reduction goals and the financial imperatives of gambling revenue. Interventions are variably perceived and implemented depending on context and framing. A lines-of-argument synthesis was used to develop an interpretation across the interventions, indicating that system-level interventions are often weakened by the economic interests of governments and the gambling industry, while individual-level interventions shift responsibility onto gamblers and staff, often resulting in limited effectiveness and unintended consequences such as migration to alternative gambling products. Limited high-quality studies per intervention, few focused on online gambling, and regional bias may affect transferability. This review underscores the need to reframe gambling harm as a systemic issue and to develop context-sensitive public policies that are clearly communicated to target populations. Effective prevention requires countering the gambling industry’s framing, which individualizes responsibility, resists regulation, and justifies availability on economic grounds, through strategies integrating systemic regulation, digital prevention tools, and harm-reduction. Policymakers should examine interventions and their assumptions, placing public health above gambling revenue.

## Introduction

Gambling is recognized as a global public health concern. Over the past decades, its availability has expanded rapidly across jurisdictions following liberalization and the rise of online gambling [1]. Gambling can lead to a range of harms, including addiction as well as adverse social, psychological, economic, and physical effects [2].

Scholars and public health experts have repeatedly called for the implementation of regulatory strategies aimed at preventing and reducing gambling harm [1,3,4]. Yet, multiple systematic reviews consistently highlight a lack of high-quality evidence supporting the effectiveness of such interventions [5–10]. A major challenge is the continued preference for individually focused strategies that emphasize personal responsibility, despite their (often) limited evidence base [8,9,11]. For example, self-exclusion is an individual-level downstream intervention that may help individuals experiencing severe gambling harm, but it depends on the affected person taking initiative to act. In contrast, upstream, population-level preventive measures—such as restrictions on the availability of gambling—are supported by stronger evidence but are frequently overlooked [11]. One suggested key reason is the close alignment of interests between governments and the gambling industry, both of which benefit financially from gambling revenues and may be disincentivized to implement more effective harm-reduction measures, as these also tend to reduce profits [11]. The urgent need to address gambling harm therefore requires both a stronger evidence base and greater emphasis on the overall effectiveness of existing interventions.

Extensive social science literature demonstrates that context shapes both the implementation and effectiveness of interventions in clinical and societal settings [12,13]. Contextual factors may influence both the effectiveness and successful implementation of preventive and harm reduction interventions across various public health domains [14]. Existing evidence suggests that factors related to both the immediate and broader implementation context—such as institutional structures, stakeholder engagement, and cultural norms—can significantly shape intervention outcomes [12]. Qualitative studies examining the implementation and outcomes of such interventions often provide rich contextual insights.

This meta-ethnographic synthesis addresses a critical gap in gambling research by examining the following research question: How contextual factors influence the implementation and effectiveness of harm reduction and prevention interventions? To our knowledge, no prior qualitative evidence synthesis has explored this question. This synthesis of qualitative research aims to identify contextual factors that affect the implementation and success of gambling harm prevention policies by synthesizing findings from qualitative studies. The purpose is ultimately to support more effective policy development, implementation, and enforcement [15].

## Materials and methods

Meta-ethnography is a method for synthesizing qualitative research evidence, originally developed by Noblit and Hare [16]. What sets meta-ethnography apart from other qualitative syntheses is its emphasis on the original data or first-order interpretations of social actors in the context—hence the allusion to meta-analysis in the method’s name. This review takes seriously Noblit and Hare’s [16] key insight that language must be understood metaphorically. Consequently, in the processes of reading, translating, and synthesizing the studies, particular attention is paid to verifying comparatively that the metaphors (interpretations) used by the authors of the primary studies refer to the same underlying phenomena.

In their seminal work, Noblit and Hare [16] introduce seven phases of meta-ethnography: getting started (identifying the topic and selecting relevant studies), reading the studies (understanding key metaphors), determining how the studies are related (listing key concepts or interpretations and their interrelations across studies), translating the studies into one another (comparing interpretations across studies to find commonalities and differences), synthesizing translations (integrating translated interpretations to create new interpretations), and expressing the synthesis (presenting the results effectively).

Given the study’s focus on how context shapes policy effectiveness and implementation, meta-ethnography was chosen for its strength in revealing meanings, interpretations, and contextual dynamics.

The review follows the eMERGe reporting guidelines for meta-ethnography [17] (S1 Table) and PRISMA guidelines for systematic reviews (S2 Table). The eMERGe guideline follows the original seven phases of meta-ethnography while offering a more robust way of reporting the synthesis. Although this meta-ethnography was not pre-registered, all methods are clearly documented to ensure rigor and reproducibility.

### Search strategy

A tailored search strategy, without restrictions on publication date, was designed to capture multidisciplinary literature across diverse databases and to accommodate database-specific indexing systems and search functionalities. The search was conducted combining subject headings related to gambling, interventions or policies (excluding studies focused solely on clinical treatment), and qualitative research with equivalent free-text terms in titles, abstracts, and author keywords. The search was conducted in Medline, PsycINFO, SocINDEX, Worldwide Political Science Abstracts (WPSA), Applied Social Sciences Index and Abstracts (ASSIA), Web of Science Core Collection, and Google Scholar. Full search strategies for each database are provided in S3 File. In addition to database searches, the journal Critical Gambling Studies—in which a lot of qualitative gambling research is published, and which is not indexed in the databases above—was manually searched. Finally, reference lists of all eligible articles were screened to identify additional studies.

The search was conducted by one author (JS), who has substantial expertise in gambling policy and qualitative research. An experienced information specialist was consulted throughout each stage of the search strategy development and execution. The final searches were completed on 3 September 2024.

### Selecting the primary studies

**Fig 1:**
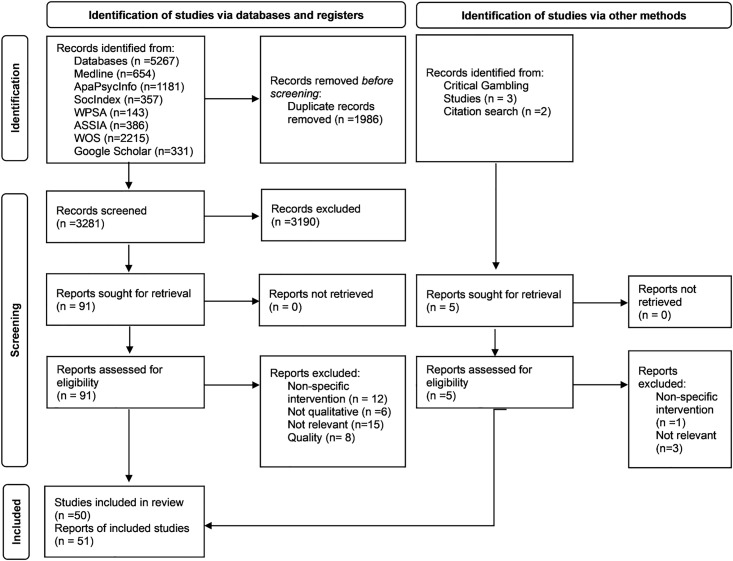
PRISMA diagram outlining the searching and screening process.

Two reviewers (JS and LK) independently screened the titles and abstracts of all retrieved records for eligibility in three steps. First, duplicates were removed. Second, the titles and abstracts were assessed against the predefined inclusion criteria: (1) original empirical studies; (2) use of qualitative methods and data; (3) focus on the implementation or effects of specific gambling prevention or harm reduction interventions, the attitudes and perspectives of stakeholders (e.g., citizens, gamblers, policymakers, regulators, industry representatives, researchers, media), or the justification and rationale of such policies; (4) publication in peer-reviewed journals; and (5) written in English. Third, the full texts of potentially relevant records were retrieved and independently assessed by both reviewers. Studies were excluded if they involved treatment and support interventions, non-specific policy interventions, were published in non-English languages, were of poor quality, or were categorized as books, book chapters, editorials, reviews, grey literature, or protocols. Any discrepancies in eligibility assessments were resolved through discussion until consensus was reached. This process resulted in 59 articles representing 58 distinct studies being retained for quality appraisal (Fig 1).

The articles were categorized into three groups (key, satisfactory, fatally flawed). Categorization was based on methodological quality, relevance and conceptual richness. Key articles were used as index articles in the synthesis due to their quality, conceptual richness and contribution [18].

A quality appraisal was conducted using the Critical Appraisal Skills Programme (CASP) checklist for qualitative research [19] (Table 1). Two reviewers (JS and LK) independently assessed the methodological quality of each study, and discrepancies in appraisals were resolved through discussion until consensus was reached. Only items that were explicitly reported were evaluated. Studies assessed as methodologically flawed or of low overall quality based on the quality appraisal were excluded from the synthesis (N = 8) [20–27], leaving 50 studies and 51 articles (Fig 1).

**Table 1:** Full CASP quality appraisal of all studies assessed for inclusion (including those excluded following quality appraisal).

	1	2	3	4	5	6	7	8	9	10
Beckett et al. 2020 [28]	Y	Y	–	Y	Y	Y	Y	Y	Y	Y
Bowne & Jarldorn 2024 [29]	Y	Y	Y	Y	Y	Y	Y	N	Y	Y
Breen et al. 2005 [20]	Y	Y	–	–	Y	–	Y	N	Y	Y
Breen et al. 2006 [21]	Y	Y	–	Y	Y	–	–	N	N	Y
David et al. 2019 [30]	Y	Y	–	Y	Y	Y	Y	Y	Y	Y
Drosatos et al. 2020 [31]	Y	Y	Y	Y	Y	–	Y	Y	Y	Y
Forsström et al. 2017 [32]	Y	Y	Y	Y	Y	Y	Y	Y	Y	Y
Forsström et al. 2022 [33]	Y	Y	Y	Y	Y	–	Y	Y	Y	Y
Forsström & Cisneros Örnberg 2019 [34]	Y	Y	–	Y	Y	–	–	Y	Y	Y
French et al. 2021 [35]	N	Y	Y	Y	Y	–	–	Y	Y	Y
Gainsbury et al. 2018a [36]	Y	Y	Y	Y	Y	–	Y	Y	Y	Y
Gainsbury et al. 2018b [37]	Y	Y	Y	Y	Y	–	Y	Y	Y	Y
Goh et al. 2016 [38]	Y	Y	Y	Y	Y	–	Y	Y	Y	Y
González Díaz et al. 2024 [39]	Y	Y	Y	Y	Y	–	Y	Y	Y	Y
Hing & Breen 2008 [22]	Y	Y	Y	Y	Y	–	–	N	Y	Y
Hing & Nuske 2011 [40]	Y	Y	Y	Y	Y	–	Y	N	Y	Y
Hing & Nuske 2012 [41]	Y	Y	Y	Y	Y	–	Y	N	Y	Y
Hing et al. 2014 [42]	Y	Y	Y	Y	Y	–	Y	Y	Y	Y
Kiyak et al. 2023 [23]	Y	Y	–	Y	Y	–	Y	N	Y	Y
Kolandai-Matchett et al. 2018a [43]	Y	Y	Y	Y	Y	–	–	Y	Y	Y
Kolandai-Matchett et al. 2018b [44]	Y	Y	Y	Y	Y	–	Y	Y	Y	Y
Korn et al. 2006 [24]	N	Y	–	Y	Y	–	Y	N	Y	Y
Kraus et al. 2023 [45]	Y	Y	Y	Y	Y	–	Y	Y	Y	Y
Lakew 2022 [46]	Y	Y	–	Y	Y	–	–	Y	Y	Y
Landon et al. 2016 [47]	Y	Y	Y	Y	Y	–	Y	Y	Y	Y
Leung & Kong 2013 [48]	Y	Y	Y	Y	Y	–	–	Y	Y	Y
Maltzahn et al. 2022 [49]	Y	N	Y	Y	Y	Y	Y	Y	Y	Y
Manian et al. 2023 [50]	Y	Y	–	Y	Y	Y	Y	Y	Y	Y
Marionneau & Järvinen-Tassopoulos 2022 [51]	Y	Y	–	Y	Y	–	Y	Y	Y	Y
Marko et al. 2023 [52]	Y	Y	–	Y	Y	Y	Y	Y	Y	Y
Marko et al. 2020 [53]	Y	Y	–	Y	Y	Y	Y	Y	Y	Y
McCarthy et al. 2022 [54]	Y	Y	Y	Y	Y	–	Y	Y	Y	Y
McCarthy et al. 2023 [55]	Y	Y	Y	Y	Y	Y	Y	Y	Y	Y
Messerlian & Derevensky 2006 [56]	Y	N	–	Y	Y	–	Y	Y	Y	Y
Messerlian & Derevensky 2007 [57]	Y	Y	–	Y	Y	Y	Y	Y	Y	Y
Miller et al. 2018 [58]	Y	Y	Y	Y	Y	Y	Y	Y	Y	Y
Morvannou et al. 2020 [25]	Y	Y	–	Y	Y	–	Y	N	Y	Y
Newall et al. 2023 [59]	Y	Y	–	Y	Y	–	Y	Y	Y	Y
Nyemcsok et al. 2022 [60]	Y	Y	Y	Y	Y	Y	Y	Y	Y	Y
Nyemcsok et al. 2023 [61]	Y	Y	–	Y	Y	–	Y	Y	Y	Y
Pickering et al. 2019 [62]	Y	Y	Y	Y	Y	Y	Y	Y	Y	Y
Pickering et al. 2022 [63]	Y	Y	Y	Y	Y	Y	Y	Y	Y	Y
Pitt et al. 2021 [64]	Y	Y	–	Y	Y	Y	Y	Y	Y	Y
Pitt et al. 2022 [65]	Y	Y	Y	Y	Y	–	Y	Y	Y	Y
Pitt et al. 2024 [66]	Y	Y	Y	Y	Y	Y	Y	Y	Y	Y
Rintoul et al. 2017 [67]	Y	Y	–	Y	Y	–	Y	Y	Y	Y
Rolando et al. 2020 [68]	Y	Y	–	Y	Y	–	Y	Y	Y	Y
Rolando et al. 2021 [69]	Y	Y	–	Y	Y	–	Y	Y	Y	Y
Selin 2016 [70]	Y	Y	Y	Y	Y	–	–	Y	Y	Y
Selin 2022 [71]	Y	Y	Y	Y	Y	–	–	Y	Y	Y
Selin et al. 2020 [72]	Y	Y	–	Y	Y	–	Y	Y	Y	Y
Selwyn et al. 2024 [26]	Y	Y	–	Y	N	–	–	–	N	Y
Swanton et al. 2023 [73]	Y	Y	–	Y	Y	–	Y	Y	Y	Y
Thomas et al. 2015 [74]	Y	Y	–	Y	Y	–	Y	Y	Y	Y
Torrance et al. 2024 [75]	Y	Y	Y	Y	Y	–	Y	Y	Y	Y
Tse et al. 2007 [27]	Y	Y	–	Y	Y	–	N	N	N	Y
van Schalkwyk et al. 2021 [76]	Y	Y	–	Y	Y	–	Y	Y	Y	Y
van Schalkwyk et al. 2022 [77]	Y	Y	Y	Y	Y	–	Y	Y	Y	Y
van Schalkwyk et al. 2024 [78]	Y	Y	–	Y	Y	–	Y	Y	Y	Y

Y = yes, N = no, - = can´t tell. In studies with JS as an author, supremacy was given to ratings of LK. (1) Was there a clear statement of the aims of the research? (2) Is a qualitative methodology appropriate? (3) Was the research design appropriate for addressing the aims of the research? (4) Was the recruitment strategy appropriate for the aims of the research? (5) Was the data collected in a way that addressed the research issue? (6) Has the relationship between the researcher and participants been adequately considered? (7) Have ethical issues been considered? (8) Was the data analysis sufficiently rigorous? (9) Is there a clear statement of findings? (10) How valuable is the research?

In addition to quality appraisal, conceptual richness of each article was assessed based on the use of concepts and analytical frameworks in reporting [18]. Further, each article was assessed in terms of relevance in relation to review aims [18].

Defining the intervention categories was a continuous, iterative process that began during the reading of the full texts and was finalized during data extraction. To enable synthesis, a minimum threshold was set: at least three articles had to provide substantial contributions to an intervention category for it to be included. Some of the articles contributed to several intervention categories. All seven intervention categories identified are well grounded in previous literature reviews [5,6,8,10,79,80]. The following categories were supported by sufficient evidence for inclusion in the meta-ethnography: educational interventions, exclusion programs, availability regulations, spending limits, advertising restrictions, behavioral interruptions, and behavioral feedback (Table 2). Of the interventions, availability regulations and advertising restrictions are system-level interventions, targeting systems and norms at the population level, while the remaining interventions focus on individual responsibility and are considered individual-level interventions [81].

**Table 2:** Policy interventions investigated.

Policy type	Description
Educational interventions	Campaigns, product information, messages, and educational materials aimed at informing specific population groups or the public about gambling risks.
Exclusion programs	Exclusion interventions include self-exclusion, where individuals voluntarily ban themselves from gambling, and third-party exclusions, such as family exclusion orders, where relatives can apply to authorities to restrict a person’s gambling.
Availability regulations	Regulatory interventions that either restrict or increase the availability and accessibility of certain gambling products to the general population.
Spending limits	Restrictions on how much individuals can stake, deposit, or lose while gambling.
Advertising restrictions	Regulations designed to protect population groups from gambling advertisements or to limit the content of such advertisements.
Behavioral interruptions	Policies aimed at encouraging or requiring individuals to stop gambling when signs of harm are detected.
Behavioral feedback	Interventions that provide individuals with feedback based on their actual gambling behavior to encourage reflection and potential behavior change.

Five articles contributed to interventions that did not fit into the employed categories [30,43,49,53,72]. Seven articles were not used in the translation phase due to their limited contributions to any of the interventions [34,44,58,60,61,64,67]. One article [56] was excluded because it reported the same results as another article by the same authors [57]. Another article [40] was excluded as it was evident that it used the same interview data as another article by the same authors and reported similar results [41].

Of the 51 eligible studies, 37 were used in the final synthesis.

### Data extraction, translation and synthesis

To assess their relevance, the articles were coded to identify all findings related to various interventions. Subsequently, excerpts from the original data, along with the corresponding interpretations by the primary authors, were extracted by LK and organized by intervention into data extraction tables [82]. All raw data excerpts (first-order interpretations) were categorized under their original themes. To explore relationships within the data, the second-order interpretations provided by the primary authors were placed in an adjacent column next to the corresponding excerpts.

The articles were compared with a focus on the review’s aims—specifically, the interventions’ effects and factors influencing their effectiveness and successful implementation. The comparison followed the detailed approach of Sattar et al. [82] and was organized by intervention. Research design was considered, alongside the primary authors’ interpretations regarding intervention effects and the identification of barriers and facilitators to implementation. Contextual factors, such as the gambling setting (online vs. land-based) and the perspectives of various demographic groups, individuals with gambling harm, regulators, treatment providers, and industry representatives were central to interpreting and relating the studies.

The studies were reciprocally related; that is, their findings were largely translatable into the terms of one another [16]. Differences in themes and second-order interpretations stemmed largely from study design, particularly aims and intervention specifics, as well as from the populations studied, with views, for example, differing between youth and adults. There was no indication of explicit refutations where interpretations of some primary authors would have been criticized by other primary authors in the light of their own interpretations [16]. There was only one clear conflict among the studies that could be considered implicitly refutational. A Canadian study on educational interventions found that young people favored responsible gambling messaging over information about product-related harms [57]. In contrast, most of the other studies included in the review were critical of responsible gambling discourse. This discrepancy may suggest that the young participants in the Canadian study had possibly internalized the dominant discourse emphasizing individual responsibility.

Primary author interpretations making up the second-order interpretations were summarized into “lists of themes” to better facilitate cross-study comparison [16,82]. In this review, themes refer to phenomena such as the effects and factors related to implementation of interventions. As most articles were descriptive, the second-order interpretations were formulated idiomatically to better enable comparisons [83]. Ensuring that these interpretations referred to comparable phenomena across studies was essential. Using the initial theme lists as a foundation, broader thematic categories were developed iteratively through reading the data extraction tables and source articles, followed by continuous refinement. Themes from individual articles were then compared and synthesized into larger cross-cutting thematic categories.

Translation tables organized by the cross-cutting thematic categories and containing first and second-order interpretations, were created [82] (Table 3). For the synthesis, the focus was on the second-order interpretations: if first-order interpretations are referred to, they are in quotation marks. Initial translation of the original articles was carried out by performing a primary data synthesis of the second-order interpretations [82] with the help of the data extraction tables and translation tables. This consisted of a systematic iterative comparison of themes in each original article. An article with key ranking and/or conceptual richness was selected as a reference article for each intervention [16]. After the initial primary data synthesis, the initial translation was tested for validity and saturation using additional articles, when possible. Additionally, synthesis tables were used [84] (Table 4; S4 File). Translations were first developed by JS and independently reviewed by LK. They were then discussed to resolve any interpretative differences and ensure consistency in the translation of interpretations.

**Table 3:** Extract from a translation table on the effects of availability policies.

Main theme	First-order interpretation	Second-order interpretation
Effects of availability changes	[My friends and I] we’re happy because if we had forty euros in our pocket, it would already be in the slots... and we couldn’t even go have a drink in the evening (...) I’ve been making it to the end of the month for two or three months now (INT. 47,Male, 56 years, area NO, PGSI:problem gambling) [69, p. 353]	A 57-year-old man, a frequent slot machine player, reported that since all EGMs^1^ had been removed from bars and tobacco shops in his small town, he finally had the means to make it to the end of the month. [69, p. 353]
	The gambler who doesn’t have problems, maybe if he doesn’t find slots all over any more, might have cut down, because the opportunity isn’t there. But when it’s a disease, when it’s a gambler who’s got an urge he can’t stop, he didn’t stop, he moved to the same sort of gaming a few kilometres away, or to another more aggressive kind of gaming that didn’t solve the problem. (NO_8, EGM provider) [67, p. 305]	The industry representatives maintained that limiting availability by introducing minimum distances can be effective in reducing betting among occasional gamblers, but not among compulsive gamblers, who would be willing to travel long distances to bet. [68]
	“a positive effect. The time I used to spend on those machines is now spent with the family” (male, 35–49). [68, p. 5]	For the vast majority, however, the closure of EGMs was a relief. Closures had reduced time and money spent gambling and improved general welfare. One respondent explained how the shutting down EGMs had had [68, p. 5]
	“started playing online when he got back from the shops” (male, 35–49). [68, p. 8]	Some had not increased their consumption, but merely changed their mode of gambling. One gambler explained that as EGMs were closed and it was not possible to play while going to the supermarket, he had [68, p. 8]

**Table 4:** An example of synthesis table on exclusion programs.

Methods and themes	Pickering et al. (2022) [63]	Pickering et al. (2019) [62]	Goh et al. (2016) [38]	Hing et al. (2014) [42]	Kraus et al. (2023) [45]
Sample	Self-excluders, counsellors, venue staff, policy makers	Current and former self-excluders	Family members of people with problem gambling	People with problem gambling (self-excluders and non-excluders)	Gamblers, venue managers, venue staff,
Data collection	Focus groups	Interview	Interview	Interview	Interview
Barriers to effectiveness		Low coverage. Difficulties in detecting self-excluders at venues.	Effectiveness undermined by the possibility to gamble outside casinos.	Availability of alternative venues. Poor monitoring. Apathy on the part of staff. Not effective.	Possibility of shifting to other gambling venues. Lack of identity checks. Low uptake. Not effective.
Facilitators and effectiveness		Treatment and support considered key component. Longer exclusion times supported. No revocation.	Family exclusion cause a sense of relief for the close ones.	Possibility to decide the length supported. Effective for some, not personally.	Gamblers considered beneficial. Staff in favor of linking additional support. Longer exclusion time supported.
Registration barriers		Registration time-consuming as one had to register to every EGM venue separately. Lack of respect by the staff. Lack of information.		Registration in a single venue difficult, and embarrassing. Negative experience of interactions with the staff. Lack of information on registration.	Staff trying to talk gambler out of the idea of self-excluding.
Registration facilitators	The emotional state of the self-excluders needs to be considered. The easiness of use. Ability use smart phone. Validating the decisions. Ability to contact help services.	Compassionate treatment. Possibility to register online may strengthen self-efficacy. Family members or other close ones play a key role. Important that the staff approaches discreetly.		Sensitivity and support from staff appreciated. Multi-venue registration. Support from help services or close ones decisive.	Approaching need to be done with right tone and time.

Reciprocal synthesis of studies related to specific interventions resulted in the creation of our own (third-order) interpretations of key insights related to the themes of each intervention [82]. In practice, primary data syntheses and translation tables were read in tandem and compared with the developed third-order interpretations (Table 5; S5 Table). Finally, based on the third-order interpretations, a lines-of-argument synthesis was constructed, integrating all the interventions analyzed [16]. Noblit and Hare [16] describe a lines-of-argument synthesis as a holistic interpretation of the studies investigated, including their relationships and contexts. A central insight that contributed to the lines-of-argument synthesis was the recurrence of certain dichotomies and conflicts of interest across interventions. These included tensions such as responsible versus irresponsible gambling, gambling revenue versus gambling harm, and divergent perspectives among key stakeholders.

**Table 5:** An example of derivation of third-order interpretations.

Intervention	Theme	Synthesis of second-order interpretations	Third-order interpretation
Exclusion programs	Facilitators of effectiveness	Treatment and support, in addition to self-exclusion, are important. No revocation of self-exclusion, longer durations, and the ability to tailor the length are supported.	Accessibility of exclusion with extensive coverage, as well as support and compassion from staff.
	Barriers to effectiveness	Bypassing by gambling in another venue, contradictions between enforcing self-exclusion and generating revenue from self-excluders, and poor monitoring.	Bypassing and lax enforcement of the exclusion hampers effectiveness.
	Registration facilitators	Online registration to multiple venues through an easy and safe site may also enhance self-efficacy. Services and close ones play an important role in the decision to self-exclude. Sensitivity, compassion, and support from staff are appreciated.	Accessibility of exclusion with extensive coverage, as well as support and compassion from staff.
	Registration barriers	Laboriousness of registration, lack of respect from staff, and feelings of shame. Lack of information about registration. The conflict between suggesting self-exclusion and the consequent loss of revenue or the risk of staff facing sanctions.	Laboriousness and distressful registration processes and companies prioritizing revenue over actively assisting gamblers.

## Results

Based on information provided in the included articles, the studies were located in the following regions: 26 (51%) from Oceania (Australia, New Zealand), 17 (33%) from Europe (Denmark, Finland, Germany, Italy, Norway, UK, Sweden), 4 (8%) from North America (Canada), 3 (6%) from Asia (Macao, Singapore), 1 (2%) from unknown location. The predominance of studies from Oceania may limit the transferability of findings to other regions, where regulatory and cultural contexts differ. The characteristics of the included studies are presented by intervention in Table 6. The syntheses are reported by intervention, with each thematic discussion concluding in our new third-order interpretations.

**Table 6:** Characteristics of the included studies.

Author, Year	Country	Participants/materials	No. of participants/materials	Data collection	Ranking	Aims
Educational interventions						
Van Schalkwyk et al. (2022) [77]	UK	Campaign materials	N/A	Secondary data	Key	To explore whether and how the resources promote a particular account of gambling and gambling harms which is amenable to industry interests.
Thomas et al. (2015) [74]	Australia	Gamblers of varying risk levels	100	Semi-structured interviews	Key	To investigate how gamblers interact with, and respond to, social marketing campaigns that focus on the risks and harms of problem gambling.
Van Schalkwyk et al. (2021) [76]	UK	Program documents	N/A	Secondary data	Key	To analyse the framing adopted by the Senet Group to conceptualize and define the problem(s) and its causes, propose solutions deemed acceptable and effective, and describe the nature and effectiveness of the campaign.
Leung & Kong (2013) [48]	Singapore	Gamblers of varying risk levels’ video testimonials form an awareness-raising campaign	4	Secondary data	Key	How the governing party in Singapore makes use of the discursively constructed juxtaposing identities of social and problem gamblers as a symbolic resource.
Torrance et al. (2024) [75]	UK, Ireland, Australia	Gambling scholars, experts by experience	12 + 10	Focus groups	Key	To evaluate a counter-advertising intervention video to increase resilience to gambling advertising persuasion.
McCarthy et al. (2022) [54]	Australia	Women aged 20–41 years	41	Semi-structured interviews	Key	To seek women’s opinions about the factors that may contribute to the normalization of gambling for women, and the strategies that may counter this normalization.
Newall et al. (2023) [59]	Australia	Academics, regulators, treatment providers	21	Focus groups	Satisfactory	To analyze the perspectives of academics, regulators, and treatment providers on designing more effective safer-gambling messages
Gainsbury et al. (2018a) [36]	Canada	Young adults, seniors, weekly gamblers, gamblers of skill-based games	39	Focus groups	Key	To understand differences between gamblers and receive qualitative feedback on targeted messages used to increase use of responsible gambling tools.
Messerlian & Derevensky (2007) [57]	Canada	Youths aged 12–18 years	175	Focus groups	Satisfactory	To explore adolescents’ exposure to existing prevention campaigns and their message content and communication strategy preferences for a youth gambling social marketing campaign.
Marko et al. (2023) [52]	Australia	Gamblers, affected others	15 + 6	Semi-structured interviews	Satisfactory	To explore how people who have experienced gambling harm interpret and apply personal responsibility frames and ‘gamble responsibly’ messages in their lives.
Pitt et al. (2022) [65]	Australia	Youths aged 11–17 years	54	Interviews	Satisfactory	To document young people’s perceptions about strategies that could be used to counter the normalization of gambling and prevent gambling harm.
McCarthy et al. (2023) [55]	Australia	Informants from research, policy, treatment and experts by experience	15 + 5	Focus groups	Satisfactory	To understand how key informants conceptualized the risks facing women who gamble, and the range of tailored strategies that could be used to respond.
Van Schalkwyk et al. (2024) [78]	UK	Program documents	N/A	Secondary data	Key	To examine whether, and how, corporate agnogenic practices are used to portray industry-funded youth education programs as effective forms of harm prevention.
Kolandai-Matchett et al. (2018b Nat)^1^ [44]	New Zealand	Public health staff; public health programs’ progress reports	8	Focus groups, secondary data	Satisfactory	To identify practical information that can be used by program planners and implementers to reinforce public health program effectiveness.
Nyemcsok et al. (2023)^1^ [61]	Australia	Male sports bettors, aged 18–24 years	16	Interviews	Satisfactory	To understand young people’s perspectives about public health promotion strategies. To provide suggestions from young male gamblers about sports betting harm prevention and reduction strategies.
Miller et al. (2018)^1^ [58]	Australia	People with present or prior experience of gambling harm from gambling machines	26	Interviews	Satisfactory	To examine the perspectives of people with lived experience of problem gambling and their implications for effective harm-reduction interventions.
Pitt et al. (2021)^1^ [64]	Australia	Adults with intellectual disability	19	Semi-structured interviews	Satisfactory	To understand the factors that may influence how and why people with intellectual disability may engage in gambling.
Selin (2022)^1^ [71]	Denmark, Finland, Norway, Sweden	Annual reports of gambling companies	12	Secondary data	Satisfactory	To find out what kind of subjects are being produced through the responsible gambling practices.
Exclusion programs						
Pickering et al. (2019) [62]	Australia	Current and former self-excluders	13 + 7	Semi-structured interview	Satisfactory	To explore consumers’ experiences of a self-exclusion program and their perspectives on its different characteristics.
Kraus et al. (2023) [45]	Germany	Gamblers, managers at venues, gambling venue staff	13 individuals, 4 groups	Interview (group & individual)	Satisfactory	To gain insight into different actors’ perceptions on the problems in the process of self-exclusion to delineate which specific attitudes hamper implementation of self-exclusion.
Hing et al. (2014) [42]	Australia	Self-excluders and non-excluders with experience of gambling harm	103	Interviews	Satisfactory	To examine how people engage with self-exclusion programs, including their motivations, barriers, experiences, perceptions, and suggestions for improvement.
Pickering et al. (2022) [63]	Australia	Stakeholders, self-excluders, gambling counsellors, venue staff, policy makers	25 + 5 + 7 + 6 + 7	Focus groups, interviews	Key	To elicit key stakeholders’ expectations of a self-exclusion website in terms of its design features and functioning; to identify practical issues that could potentially impact the website development and implementation.
Goh et al. (2016) [38]	Singapore	Family members of people experiencing gambling harm	105	Interviews	Satisfactory	To illuminate the issues experienced by family members that provided the impetus for them to apply for the family exclusion order.
Bowne & Jarldorn (2024)^1^ [29]	Australia	Venue staff	5	Interviews	Satisfactory	To explore venue employees’ perceptions and experiences of implementing responsible gambling measures.
Rintoul et al. (2017)^1^ [67]	Victoria, Australia	Gamblers, professionals	40 + 20	Observations, interviews, focus groups, secondary data	Satisfactory	To explore whether practices relating to responsible gambling are actually implemented in local club and hotel venues.
Availability regulations						
French et al. (2021) [35]	Canada	Documents, media, ethnographic fieldnotes	N/A	Secondary data, ethnographic field observation	Satisfactory	To examine internet gambling website blocking scheme, the article illustrates the complexities of regulating online gambling.
Rolando et al. (2020) [68]	Italy	Stakeholders	30	Focus groups	Satisfactory	To provide a picture of the current debate on gambling regulation.
Marionneau & Järvinen-Tassopoulos (2022) [51]	Finland	Gamblers	187 + 27	Interviews, questionnaire	Satisfactory	How past-year gamblers experience prolonged closures of gambling machines and occasional re-openings one year into the COVID-19 pandemic.
Rolando et al. (2021) [69]	Italy	Gamblers	60	Semi-structured interviews	Satisfactory	To investigate gamblers’ opinions and perspectives on why and how the new regulation impacted their habits.
McCarthy et al. (2022)^1^ [54]	Australia	Women aged 20–41 years	41	Semi-structured interviews	Satisfactory	To seek women’s opinions about the factors that may contribute to the normalization of gambling for women, and the strategies that may counter this normalization.
McCarthy et al. (2023)^1^ [55]	Australia	Informants from research, policy, treatment and experts by experience	15 + 5	Focus groups	Satisfactory	To understand how key informants conceptualized the risks facing women who gamble, and the range of tailored strategies that could be used to respond.
Miller et al. (2018)^1^ [58]	Australia	People with present or prior experience of gambling harm from gambling machines	26	Interviews	Satisfactory	To examine the perspectives of people with lived experience of problem gambling and their implications for effective harm-reduction interventions.
Nyemcsok et al. (2022)^1^ [60]	UK	Gamblers (aged 29–60 years) with experiences of gambling harm	20	Semi-structured interviews	Satisfactory	To explore how experts by experience define priorities for preventing and reducing gambling harm and identify barriers and facilitators to their engagement in policy development.
Pitt et al. (2022)^1^ [65]	Australia	Youths aged 11–17 years	54	Interviews	Satisfactory	To document young people’s perceptions about strategies that could be used to counter the normalization of gambling and prevent gambling harm.
Spending limits						
Gainsbury et al. (2018b) [37]	Australia	Regular machine gamblers	31	Focus groups	Key	To investigate the best way to introduce pre-commitment tool for gambling machines that will maximize the perceived value and subsequent uptake by gamblers.
Lakew (2022) [46]	N/A	Users of online deposit limits	10	Interviews	Satisfactory	To report on the implementation, at the payment-solution level, of an intervention tool that gambling customers used to deposit their bets.
Swanton et al. (2023) [73]	Australia	Machine gamblers, aged 24–76 years	26	Focus groups	Satisfactory	To explores the perspectives of gamblers regarding the concept of cashless gambling.
Drosatos et al. (2020) [31]	UK, Europe	Addiction experts and gamblers	13 + 6	Semi-structured interviews	Satisfactory	To explore the range of data and modalities of interaction which can facilitate richer interactive persuasive interventions and offer additional support to limit setting.
Selin (2022) [71]	Denmark, Finland, Norway, Sweden	Annual reports of gambling companies	12	Secondary data.	Satisfactory	To find out what kind of subjects are being produced through the responsible gambling practices.
McCarthy et al. (2022)^1^ [54]	Australia	Women aged 20–41 years	41	Semi-structured interviews	Satisfactory	To seek women’s opinions about the factors that may contribute to the normalization of gambling for women, and the strategies that may counter this normalization.
Miller et al. (2018)^1^ [58]	Australia	People with present or prior experience of gambling harm from gambling machines	26	Interviews	Satisfactory	To examine the perspectives of people with lived experience of problem gambling and their implications for effective harm-reduction interventions.
Nyemcsok et al. (2022)^1^ [60]	UK	Gamblers (aged 29–60 years) with experiences of gambling harm	20	Semi-structured interviews	Satisfactory	To explore how experts by experience define priorities for preventing and reducing gambling harm and identify barriers and facilitators to their engagement in policy development.
Nyemcsok et al. (2023)^1^ [61]	Australia	Male sports bettors, aged 18–24 years	16	Interviews	Satisfactory	To understand young people’s perspectives about public health promotion strategies. To provide suggestions from young male gamblers about sports betting harm prevention and reduction strategies.
Pitt et al. (2022)^1^ [65]	Australia	Youths aged 11–17 years	54	Interviews	Satisfactory	To document young people’s perceptions about strategies that could be used to counter the normalization of gambling and prevent gambling harm.
Advertising restrictions						
González Díaz et al. (2024) [39]	Sweden	Representatives of government, industry, media, and mutual support groups. Document and media material.	12	Semi-structured interviews, secondary data	Key	To examine how stakeholder groups perceived and interpreted the key concept of moderation.
Pitt et al. (2022) [65]	Australia	Youths aged 11–17 years	54	Interviews	Satisfactory	To document young people’s perceptions about strategies that could be used to counter the normalization of gambling and prevent gambling harm.
Pitt et al. (2024) [66]	Australia	Youths aged 12–17 years	64	Focus groups	Key	To explore young Australians’ perceptions of current policy responses to gambling advertising.
Selin (2016) [70]	Finland	Annual reports of gambling companies, official public documents	N/A	Secondary data	Satisfactory	To analyse how the relationship between responsible gambling programs, and state regulation has changed.
McCarthy et al. (2022) [54]	Australia	Women aged 20–41 years	41	Interviews	Satisfactory	To seek women’s opinions about the factors that may contribute to the normalization of gambling for women, and the strategies that may counter this normalization.
Nyemcsok et al. (2022)^1^ [60]	UK	Gamblers (aged 29–60 years) with experiences of gambling harm	20	Semi-structured interviews	Satisfactory	To explore how experts by experience define priorities for preventing and reducing gambling harm and identify barriers and facilitators to their engagement in policy development.
Nyemcsok et al. (2023)^1^ [61]	Australia	Male sports bettors, aged 18–24 years	16	Interviews	Satisfactory	To understand young people’s perspectives about public health promotion strategies. To provide suggestions from young male gamblers about sports betting harm prevention and reduction strategies.
Behavioral interruptions						
Beckett et al. (2020) [28]	NSW, Australia	Venue managers and staff	20	Focus groups	Satisfactory	To understand experiences and attitudes towards existing responsible gambling training programs.
Hing & Nuske (2012) [41]	Queensland, Australia	Venue staff	48	Semi-structured interviews	Satisfactory	To explore the challenges venue employees, face when: (1) a patron directly requests assistance, (2) a patron shows signs of problem gambling without requesting help, and (3) a family member reports the patron’s gambling problem.
Manian et al. (2023) [50]	Macao	Venue staff	49	Semi-structured interviews	Satisfactory	To examine venue employees’ perceptions of responsible gambling, how they detect problem gamblers, and what barriers are impeding them from acting.
Bowne & Jarldorn (2024) [29]	Australia	Venue staff	5	Interviews	Satisfactory	To explore venue employees’ perceptions and experiences of implementing responsible gambling measures.
Landon et al. (2016) [47]	New Zealand	Gamblers of varying risk levels, gambling venue staff	40 + 19	Focus groups	Satisfactory	To document the views and experiences of popup messages from gamblers and venue staff.
Rintoul et al. (2017)^1^ [67]	Victoria, Australia	Gamblers, professionals	40 + 20	Observations, interviews, focus groups, secondary data	Satisfactory	To explore whether practices relating to responsible gambling are implemented in local club and hotel venues.
Selin (2022)^1^ [71]	Denmark, Finland, Norway, Sweden	Annual reports of gambling companies	12	Secondary data	Satisfactory	To find out what kind of subjects are being produced through the responsible gambling practices.
Behavioral feedback						
Forsström et al. (2017) [32]	Sweden	Gamblers	20	Semi-structured interviews	Satisfactory	To investigate users’ views and experiences of the responsible gambling tool Playscan.
Landon et al. (2016) [47]	New Zealand	Gamblers of varying risk levels, venue staff	40 + 19	Focus group	Satisfactory	To document the views and experiences of popup messages from gamblers and venue staff.
Swanton et al. (2023) [73]	Australia	Machine gamblers, aged 24–76 years	26	Focus groups	Satisfactory	To explores the perspectives of gamblers regarding the concept of cashless gambling.
Drosatos et al. (2020) [31]	UK, Europe	Addiction experts and gamblers	13 + 6	Semi-structured interviews	Satisfactory	To explore the range of data and modalities of interaction which can facilitate richer interactive persuasive interventions and offer additional support to limit setting.
Forsström et al. (2022) [33]	Norway	Gamblers	757	Web questionnaire with an open-ended question	Satisfactory	To explore gamblers’ perception of their risk assessment in the responsible gambling tool Playscan.
Selin (2022) [71]	Denmark, Finland, Norway, Sweden	Annual reports of gambling companies	12	Secondary data	Satisfactory	To find out what kind of subjects are being produced through the responsible gambling practices.
Lakew (2022)^1^ [46]	N/A	Users of online deposit tool	N/A	Interviews	Satisfactory	To report on the implementation, at the payment-solution level, of an intervention tool that gambling customers used to deposit their bets

^1^Not in focus of translations due to minor contribution.

### Educational Interventions

#### Effects and unintended effects of educational material.

A major theme was that, while existing educational messages and campaigns by governments and industry are often represented as effective, they are largely considered ineffective in contributing to behavioral change in gamblers and to the reduction and prevention of gambling harm [52,54,59,65,76,78]. The emphasis on individual responsibility evident in many campaigns is considered a factor that decreases the effectiveness of educational messages and campaigns, seeking instead to produce subjects who uncritically accept the existing situation, where individual responsibility is presented as the key solution to gambling harm [65,55,77].

One effect of messaging with a focus on individual responsibility is the production of feelings of shame in people experiencing severe gambling harm, which may also lead to avoidance of help-seeking [59,74]. To be effective, young people in school, gamblers and their close ones demand the inclusion of warnings on the risks of gambling products in educational messages [57,52,54,65,55,74]. Some school children preferred messages with a focus on personal responsibility to the kinds of warning messages used in other public health campaigns (e.g., smoking) [57]. A suggested improvement was also tailoring messages by risk level and demography [59,55].

Our interpretation is that the industry portrayal of individuals as both the cause and solution to gambling harm conflicts with other stakeholders’ calls for developing more effective educational interventions. There was little refutational evidence presented in the studies included on the theme of effect and effectiveness of educational interventions.

#### The figures of responsible and irresponsible gamblers.

Across studies and jurisdictions, a theme to be noted was the focus on the figures of responsible and irresponsible gamblers. The dichotomy between the responsible or rational gambler who practices self-control while gambling and the implied irresponsible or irrational gambler who belongs to the vulnerable minority lacking self-control is produced and reproduced in educational messages and campaigns [54,76,77,48]. Instead of reproducing and reinforcing the figure of the responsible gambler, it was suggested that citizens should be provided with more precise information on gambling harm and the risks of gambling products [54].

Our interpretation is that the dichotomy between the common rational gambler and the uncommon irresponsible gambler highlights that in much of the educational materials, solutions to gambling harm are to be found at the individual level. This way of representing gambling harm is at the heart of the politics of gambling and responsible gambling.

#### The structure and style of messages and videos.

As a minor theme, the studies included ways to improve the content and structure of educational messages on different channels. Overall, simple language is preferred, along with concise content and avoiding an accusing tone [36,75]. People experiencing gambling harm did not find the responsible gambling messages appealing or relevant to themselves, compared to the positive promotional messages [74].

The interpretation of this theme is mainly about improving the content and style of educational messages. The messages need to be straightforward, non-accusing, and more positive, to match up with the positivity of marketing messages.

### Exclusion programs

#### Effects and effectiveness of self-exclusion or family exclusion order.

Several barriers to the effective implementation of exclusion programs were identified across studies and jurisdictions. Having to apply for self-exclusion for oneself or on behalf of a family member separately for each individual venue was considered a major difficulty by both gamblers and their close ones [38,42,45,62]. Consequently, bypassing or circumventing an existing exclusion becomes easy, reducing the intervention’s effectiveness. Relatedly, gamblers considered that monitoring or enforcing the self-exclusions at venues was often poorly executed, likewise decreasing the effectiveness of the intervention [42,62]. Moreover, many gamblers and gambling venue staff were generally critical of the implementation of self-exclusion programs and did not consider them effective or beneficial [42,45].

Overall, self-exclusion was considered beneficial by both gamblers [42,45] and their close ones who had applied for family exclusion [38]. Self-excluders and venue staff members viewed treatment and support as important in complementing a self-exclusion program [45,62]. In addition, longer self-exclusion periods with restricted revocation were supported by gamblers, especially for people experiencing serious gambling harm. The idea of tailoring the length of the exclusion period to the needs of the individual was also supported by gamblers [42,45,62]. Some venue staff supported an unlimited self-exclusion period for people with gambling problems, while others opposed this [45].

Overall, exclusion programs may have benefits that can be enhanced with additional support and the possibility to tailor the length of the exclusion without too easy revocation. Conversely, the ease of bypassing the exclusion via alternative gambling opportunities, and lax enforcement hamper its effectiveness.

#### Registration barriers and facilitators.

Similarly to the overall effects of exclusion programs, self-excluders saw registration as too time-consuming and requiring resources, as one needs to register separately at every electronic gambling machine venue [42,62]. Improving the ease of registration with the possibility of multi-venue self-exclusion and easy-to-use online registration by, e.g., a mobile phone application, were supported to correct this [42,62,63].

Self-excluders and non-excluders reported several barriers to registration: shame and concerns over staff attitudes, negative experiences with staff, staff trying to talk customers out of the idea of self-excluding, and lack of information or staff knowledge [42,45,62]. As a corrective measure, gamblers suggested that staff should encounter potential self-excluders in a way that takes their emotional state into consideration [42,45,62,63]. Family members or other close ones often played an important role in the decision to self-exclude [38,42,62,63]. An additional challenge for registration may be that gambling venues are seen as socially important meeting places [42,45]. According to some self-excluders, denial of their own problems was an additional reason for not self-excluding [42,45].

Accessibility of exclusion with extensive coverage, as well as support and compassion from staff, facilitate registration to self-exclusion programs. In contrast, the overall laborious and distressful registration processes can be a major barrier to self-exclusion. Moreover, there is evidence of gambling companies prioritizing revenue over actively assisting and approaching gamblers at venues, possibly preventing the inclusion of many people experiencing gambling harm from self-exclusion programs.

### Availability regulation

#### Effects of availability changes.

Effects of restrictions constituted a major theme. In Italy, industry representatives and business owners accommodating gambling machines emphasize the negative financial consequences of such restrictions [68]. In Finland, some casual gamblers reported a negative impact on their enjoyment [51]. However, concerns about migration to other gambling products—such as online gambling, illegal gambling, or gambling in regions without restrictions—were frequently cited as a cross-cutting consequence of availability restrictions, especially by representatives of the gambling industry and associated businesses [51,68,69]. Gamblers reported mixed effects of migration to other gambling products, with some experiencing increased spending and harm, while others benefited from online gambling features, such as the ability to set spending limits [51,69].

Other perceived effects of availability restriction related to gambling harm were also reported. Gamblers experiencing varying levels of gambling harm reported that reduced availability of gambling machines had a positive impact, including lower expenditure, fewer harms, reduced distress, and prevention of habitual gambling [51,69].

Conversely, perceived effects of increased availability included higher spending and greater harm, as reported by both gamblers and other stakeholders, such as small business owners accommodating gambling machines [68,51].

Overall, our interpretation suggests that changes in availability may be linked to experiences of harm and changes in individual expenditure. Moreover, perspectives and arguments on the effects of availability regulation vary significantly based on stakeholders’ positions. The gambling industry and its collaborators argue that availability regulations lead to revenue loss and, most importantly, migration to alternative gambling products or formats. This bypassing or circumvention of restrictions is often presented as rendering availability restrictions ineffective.

#### Public health and economic justification of availability regulation.

The minor theme shows that both economic and public health arguments are used to justify availability restrictions and liberalizations [68,35]. In Quebec, the government justified restricting offshore online gambling as a public health measure, though the true intent seemed to be to protect the state monopoly and secure revenue [35]. The government’s expansion of gambling machines is financially justified, with Italy’s economic crisis pushing businesses to install them and financially strained individuals to gamble for income or escapism [68].

The fluidity of economic justifications used by governments and other key actors both to support and oppose availability restrictions and liberalizations poses a challenge for prevention and public health. This highlights the (often problematic) dual role of government as both the regulator of gambling operations and their direct beneficiary via taxes or ownership in gambling companies, even as these operations produce harm to the very people the government is meant to represent [85].

### Advertising restrictions

#### Interpretation of law, regulation, self-regulation and the free market.

This major theme covers the dynamics related to the gambling industry, media, government, and other stakeholders in influencing how gambling marketing is regulated or not regulated. The industry and media emphasize challenges in interpreting the meaning of “moderation” in Swedish law, necessitating the wait for case law, while governmental authorities see the interpretation as clear [39]. In Finland, while the industry and regulator disagreed on the interpretation of the marketing law, the regulator’s view prevailed, unlike in risk assessments where companies could invoke their own self-regulatory codes [70].

The industry and media argue that regulation is not needed, as the self-correcting force of the market will naturally decrease the volume of gambling marketing. Arguments against restrictions based on principles of freedom of trade, freedom of expression, and democracy were also expressed [39]. However, when the regulator established an interpretation of the law, it maintained that the focus of marketing needs to be on channeling existing demand toward legal supply without creating new demand [70].

Self-regulation and revenue priorities dominate government, industry, and media agendas. The industry views restrictions as a competitive threat, while harm prevention groups see all marketing as excessive and doubt industry compliance [39]. Australian youth acknowledge the complexity of marketing restrictions and highlight the government’s conflicted role as both regulator and revenue recipient. They also see insufficient action and awareness around gambling harm, despite recognizing the need to regulate marketing across media [65,66].

Given the differing views of the gambling industry, media, and other stakeholders, careful definition of content and interpretation of marketing restrictions is crucial to limit the exposure of various population groups, such as young people, to the potentially harmful impacts of gambling promotion. Without clear legislation, strong industry opposition to regulation may prevail due to their business interests and preference for self-regulatory alternatives.

#### Effects and impacts of marketing as justifications of restrictions.

A minor theme was that there are many different perceived effects of gambling marketing. These effects were used as justifications for restrictions on marketing. One response is annoyance over exposure due to the overwhelming volume of advertisements in different channels and environments [39]. Prolonged exposure to advertisements may also affect young people’s attitudes and decisions [66]. Moreover, having promotional and warning content in the same messages was perceived as contradictory by young people [65]. Women also recognized that gambling companies seek to appeal to them with specific products and commercial content, inciting them to gamble more [54].

Thus, it is possible that marketing messages may impact the attitudes and behavior of different population groups, justifying the need to restrict gambling advertising.

#### Consumer protection.

Restricting gambling advertisement content and protecting consumers emerged as a subtheme. State-controlled operators favored categorizing products by risk, while private online firms preferred categorizing gamblers, reflecting their reliance on high-risk online gambling revenue [39]. Young people exposed to pervasive advertising stressed the need for clear risk information and youth engagement in policy design [65,66]. Women also supported marketing restrictions to protect children [54].

Private gambling companies may oppose regulations on the content of advertisements. Still, warnings about the risks of gambling could be included in gambling marketing to counter their positive content and effects. Engaging target groups in designing marketing restrictions and the content of counter-messaging is important, as they may have experiences and insights that policymakers and officials do not know.

### Behavioral feedback

#### Responses, perceived effects, and usefulness of the feedback.

Main theme on the views on the benefits and effects of behavioral feedback showed variety across studies. While feedback could be beneficial for some gamblers to a limited extent or enable a change of focus, it was generally not considered a significant contributor to behavioral change [31–33,47,73]. Overall, both staff and gamblers, including those experiencing gambling harm, at gambling machine venues did not consider automated mandatory feedback effective in changing behavior [47]. Gamblers and staff found the feedback distracting, frustrating, and annoying [31,47]. However, some machine gamblers found the feedback helpful in maintaining control and keeping track of spending [73]. A feedback tool was considered helpful, especially for t on personal expenditure over time was supported and considered useful by gamblers [31,73].

Feedback may enable a change of focus, but generally, gamblers question its relevance and may even find mandatory feedback annoying and frustrating. The feedback is often not considered relevant to oneself, as it is perceived that people experiencing severe gambling harm are the primary target group.

#### Privacy concerns, content, and accuracy of the feedback.

The accuracy, validity, and content of the feedback were sometimes contested, given differences in income or the possibility of several people using the same account. Sometimes the feedback was considered nonsensical, accusatory, or even insulting [32,33]. Still, some online gamblers found behavioral feedback easy to read and grasp [32]. Some gambling machine venue staff considered feedback messages intrusive and were also concerned about increasing institutional control and monitoring [47,73]. Privacy concerns and embarrassment arose from the potential visibility of feedback content to others, as well as concerns over access to sensitive player data [47,73].

The implementation of personal behavioral feedback may be perceived by gamblers as governmental or gambling industry monitoring and control or as an intrusion into personal freedom. Moreover, as behavioral feedback is ultimately based on collecting behavioral data, the storage and safety of that potentially sensitive data can be a privacy concern. Additionally, if the tone of the feedback is interpreted as accusatory, it may arouse negative emotions and be counterproductive to the aim of preventing and reducing gambling harm.

### Behavioral interruptions

#### Barriers to interrupting gambling of customers at venues.

A major theme across studies and jurisdictions was the tension experienced by staff between being employees in a profit-seeking gambling business and the demands of caring for the wellbeing of customers. This was conceptualized in the second-order interpretations as role conflict or role ambiguity—the challenge of caring for customers’ welfare while fearing loss of revenue, sanctions from employers, or loss of personal income if approaching customers proactively [41,28,29,50]. Staff in gambling machine venues and casinos often rationalized their reluctance to intervene upon seeing potentially harmful behavior due to a lack of training and experience required to identify signs of gambling harm [28,29,50]. Moreover, not knowing the financial or overall situation of the customer was a reason not to approach them [41,28,50]. Additionally, staff considered existing legislation and industry codes of conduct as not allowing proactive approaches to customers, including concerns over legal repercussions if staff unintentionally offend customers [41,28,50]. Fear of angry customer responses and complaints also emerged as a common concern [41,28,50].

In contrast, management rationalized abstinence from intervening by pointing to the availability of support and self-exclusion information at the venue [28]. Additional barriers for staff included customers’ refusal to acknowledge a possible problem or ignoring the presence of the employee [41,50]. Sometimes staff members felt that intervening represented an invasion of customers’ privacy or found it emotionally laborious to face a customer clearly experiencing gambling harm [41].

Challenges to interrupting risky behavior include the responsibility placed on frontline staff to manage emotionally charged encounters with gamblers experiencing harm. This transfer of responsibility to frontline venue staff refers to the ways employees are “walking on a tightrope” [29] when trying to balance the conflicting demands of extracting revenue from high-spending gamblers experiencing harm and the duty to care for their wellbeing. Care for customer welfare, business profits, and employee income often come into direct conflict. Ultimately, staff carry the burden of implementing the policy of interrupting harmful gambling behavior.

#### Responses and effectiveness of automated and human interruptions.

A minor theme related to how interruptions in gambling are perceived and responded to by gamblers. Managers at venues viewed customer support procedures as clear and effective [28]. However, staff criticized automated alarm systems as ineffective, noting that behavioral interventions rarely followed the alerts [29]. Interruptions were also absent even when revenue spikes and overspending were evident [50].

Overall, both automated and human interruptions of risky behavior at venues were largely considered ineffective by gamblers and staff. Some managers of machine gambling venues considered practices based on existing codes of conduct functional. Managers, perhaps more strongly representing the business interests of the venue, foreground the functionality of existing policies, while staff implementing the policies in practice see them as inadequate.

### Spending limits

#### Effects and impact of spending limits on self-control, harm, and user experience.

Gamblers with different levels of risk generally considered spending limits effective and beneficial in reducing expenditure and controlling gambling across jurisdictions [31,73,37,46,71]. Moreover, extending spending limits across venues and sites was seen as increasing user value, uptake, and preventing the possibility of circumventing the limits [73,37,46]. However, some gamblers did not find spending limits and related player identification useful, believing such interventions would take the enjoyment out of gambling, result in stopping gambling, or lead to migration to other gambling products [73,37].

Overall, studies indicate that gamblers perceive potentially beneficial effects on spending and self-control. Spending limits help maintain control over gambling and provide a sense of safety while gambling. Universal limits without the option to circumvent them would potentially enhance effectiveness. However, gamblers have important reservations, viewing spending limits as reducing enjoyment, or not being relevant or beneficial to themselves, but only to others. Migration to other gambling products, such as online gambling, was considered a possibility with mandatory spending limits.

#### Issues with privacy, stigma, and freedom.

As a minor theme, privacy concerns were raised about who has access to data and how it may be used by the government or gambling industry. People experiencing gambling harm regarded the use of identification cards at venues in front of other customers as stigmatizing [37]. Mandatory spending limits were viewed as restrictions on personal freedom, raising concerns about government and industry monitoring and misusing player data [73].

Spending limits require personal identification and are potentially considered as surveillance and control by the gamblers, with the suspicion that data is collected and used data against players’ interests. These concerns were notably expressed by machine gamblers in Australia, indicating that gambling is seen there notably as an activity belonging to the private sphere rather than a public health issue.

#### Easy and flexible use.

Another minor theme was related to the way gamblers emphasized the need for a spending limit scheme that is easy to use and flexible in setting and adjusting personal limits, even on short notice and without delay [73,37,46]. Representing and implementing the spending limit scheme in a fun and light way was also supported [37].

Our interpretation is that for many gamblers flexibility and user-friendliness in setting limits were core values. However, the apparent difficulty of linking these wishes with the aims of preventing and reducing gambling harm effectively was not addressed by the participants.

### A lines-of-argument synthesis across interventions

This meta-ethnography employs a line of argument synthesis to provide based on the third-order interpretations a general interpretation of the effects, as well as the contextual barriers and facilitators influencing the implementation of the interventions. Three overarching propositions can be presented based on the third-order interpretations (S6 File):

1. The framing, perceived effects, and effectiveness of interventions vary, and the interventions can have unintended consequences.
2. Addressing gambling harm effectively involves countering the gambling industry’s influence and its framing of both the causes and the solutions to gambling harm.
3. Lax enforcement and the shifting of responsibility to frontline personnel may result in neglecting the specific needs of individuals affected by gambling harm.

There is a core tension in gambling harm prevention interventions: the conflict between economic imperatives of gambling revenue and the goal of reducing and preventing gambling harm. The tension between gambling revenue and gambling harm prevention shapes how the industry frames gambling harm and prevention, emphasizing individual responsibility and downplaying structural causes.

This tension shapes the division between system-level interventions (advertising restrictions and availability regulation) and individual-level interventions (e.g., behavioral feedback, exclusion programs, and behavioral interruptions). System-level interventions are often weakened by government and industry interests in revenue, while individual-level interventions shift responsibility onto staff and individuals, further individualizing harm reduction. Responsibility for harm reduction is often shifted onto frontline staff, who face conflicting demands between customer care and revenue generation. Consequently, both types of interventions are not only variably perceived and enacted but also can have unintended consequences that limit their effectiveness.

These structural influences extend beyond policy design to shape how interventions are experienced. Gamblers’ skepticism toward some interventions can be understood as a reaction to systemic factors, such as conflicting economic and public health goals and the individualized framing of gambling harm. The delegation of responsibility for gambling harm reduction and prevention to frontline staff burdens the staff with emotionally complex encounters. As a result, interventions on the venue level often fail to address the specific needs of individuals experiencing harm.

## Discussion

The aim of the meta-ethnography was to identify contextual factors influencing the effectiveness and implementation of harm prevention and reduction policies.

The results reveal how educational interventions often reinforce an individualistic framing of gambling harm, portraying the responsible gambler as rational and the problem gambler as irrational. This framing conflicts with other stakeholders’ calls for more effective solutions, rather than responsible gambling messages, which are considered ineffective. More effective, tailored interventions targeting adolescents with different risk profiles could be implemented in both school and online settings, with a focus on strengthening future-oriented thinking [86].

For exclusion programs, effectiveness is undermined by barriers like complex registration processes, lax enforcement, and industry prioritization of revenue over support. Easier access to alternative gambling options further limits the impact of exclusion programs.

Overall, evidence supports the effectiveness of availability restrictions although stakeholder perspectives vary. Industry perspectives often highlight migration to alternative products and economic losses.

Advertising restrictions face challenges due to industry and media resistance, coupled with self-regulatory preferences which complicated implementation. However, marketing messages may impact behavior, justifying restrictions.

Feedback interventions are met with skepticism by gamblers, who question their relevance and effectiveness and see them as intrusive. Privacy concerns arise from the collection of data and perceived surveillance.

Behavioral interruptions place a heavy burden on frontline staff, caught between the demands of revenue and duties related to the welfare of the customers. Automated and human interruptions are both often viewed as ineffective.

Spending limits are seen by some as helpful for maintaining self-control and safety but also as reducing enjoyment and having limited personal relevance. The potential value of spending limits can be understood through the lens of the prevention paradox, which holds that most gambling harm occurs among the far larger group of low- and moderate-risk gamblers rather than among high-risk gamblers [87]. Accordingly, population-level measures such as spending limits and availability restrictions may substantially reduce overall harm, even if they have limited impact on the gambling behavior of those with the highest severity. Moreover, privacy, stigma, and surveillance concerns emerge, and many gamblers value flexibility and user-friendliness.

The findings on the effects of availability regulations align with the evidence supporting their effectiveness [6,11], while educational interventions were considered largely ineffective by most studies, consistent with existing evidence [11]. The division between rational and irrational consumers, evident in the research on educational interventions, is often the point of departure for gambling policies, camouflaging the apparent conflict between the economic imperative of producing gambling revenue for the “government-industry complex” [88] and the protection of populations from gambling harm [89,90].

Although there is fairly good evidence for the effectiveness of exclusion programs, spending limits, and behavioral feedback [8], the results suggest that implementation context significantly influences their success, with bypassing exclusion programs posing a key barrier [91]. The low uptake of voluntary spending limits, noted in the literature [92], may stem from concerns about personal privacy and freedom, which also emerged in relation to behavioral feedback. Advertising restrictions face strong opposition from the gambling industry, reflecting the industry’s resistance to population-level measures due to perceived harm to business [93]. Additionally, the conflict between revenue generation and harm reduction is particularly pronounced with behavioral interruptions at gambling venues, as employees face role conflicts—a finding relevant not only to land-based gambling but also to online gambling environments. These insights highlight the importance of engaging target groups and clearly communicating the purpose and implementation of interventions in policy design and implementation.

The line of argument synthesis offers a cross-cutting interpretation of the results concerning individual interventions. The tension between gambling revenue and public health goals shapes how harm is framed and addressed. System-level interventions are weakened by financial motives, while individual-level approaches shift responsibility onto staff and gamblers, leading to limited effectiveness and unintended consequences. Overall, the way interventions are framed can influence how they are perceived and experienced, potentially affecting their effectiveness and leading to unintended consequences, such as migration to other gambling products.

Moreover, the line-of-argument synthesis suggests that conflicts of interest exist at multiple levels. Governments often face a dual role as both regulators and beneficiaries of gambling revenues [11]. Local authorities may also struggle between revenue generation, harm prevention, and alignment with national policies [68,69]. Furthermore, the gambling industry’s inherent conflict of interest between harm prevention and profit generation is widely acknowledged in the literature [94]. Gambling industry employees may experience ethical stress when job security and income conflict with harm-reduction efforts, creating barriers to the implementation and effectiveness of such interventions. Future research should examine how these conflicts influence policy and intervention outcomes.

This synthesis is limited by the small number of high-quality studies per intervention (except for educational interventions), the overrepresentation of studies from Oceania, and the limited focus on digital gambling environments, all of which may affect the transferability of the findings. However, the inclusion of diverse regions and participant groups across intervention categories helps mitigate these biases.

The research team had expertise in gambling policy and qualitative methods, supported by an information specialist. While methodological guidance on meta-ethnography is abundant and of high quality, its application to public health interventions or policies remains limited. Consequently, the established methodology required adaptation to suit the specific focus of this study on the contextual settings and effects of various interventions.

## Recommendations and conclusions

To effectively prevent gambling harm, public health strategies must challenge the gambling industry’s dominant framing, which individualizes responsibility, resists regulation, and justifies availability through economic arguments. These framings obscure structural causes and undermine population-level solutions. Reframing harm as a systemic issue is essential to shift the focus from individual behavior to wider structural determinants and to apply multi-level strategies integrating regulation, digital prevention tools, and harm-reduction frameworks. This synthesis further highlights the need to critically examine not just the design of interventions, but also the narratives and assumptions that underpin them.

## Supporting information

S1 Table The eMERGe reporting guideline checklist. (DOCX)

S2 Table PRISMA 2020 Checklist. (DOCX)

S3 File Database searches. (DOCX)

S4 File Synthesis tables. (DOCX)

S5 Table Second and third order interpretations by intervention. (DOCX)

S6 File Line of argument contributions of the review articles. (DOCX)
