# Supplementary material for: Context matters: A meta-ethnography investigating barriers and facilitators for the effective implementation of gambling harm prevention and reduction policies
Source: PLoS One. 2026 Feb 25;21(2):e0343595. doi: 10.1371/journal.pone.0343595 (PMC12935266; doi:10.1371/journal.pone.0343595)
Supplement: S1 Table — (DOCX) [file pone.0343595.s001.docx]

**S1 Table. The eMERGe reporting guideline checklist.**

| Phase | Item | Reporting criteria | Criteria met (Page No) |
| --- | --- | --- | --- |
| **Phase 1: Selecting meta-ethnography and getting started** | | | |
| *Introduction* | | | |
| 1 | Rationale and context for the meta-ethnography | Describe the gap in research to be filled by the meta-ethnography, and the wider context of the meta-ethnography | Page 3–4 |
| 2 | Aim(s) of the meta-ethnography | Describe the meta-ethnography aim(s) | Page 4 |
| 3 | Focus of the meta-ethnography | Describe the meta-ethnography review question(s) (or objectives) | Page 4 |
| **Phase 2: Deciding what is relevant** | | | |
| *Methods* | | | |
| 4 | Rationale for using meta-ethnography | Explain why meta-ethnography was considered the most appropriate qualitative synthesis methodology | Page 5 |
| 5 | Search strategy | Describe the rationale for the literature search strategy | Page 5–6, S2 Appendix 2 |
| 6 | Search processes | Describe how the literature searching was carried out and by whom | Pages 5–6 |
| 7 | Selecting primary studies | Describe the process of study screening and selection, and who was involved | Pages 6–11, Table 1 |
| 8 | Outcome of study selection | Describe the results of study searches and screening | Page 7–11, Fig. 1, Table 2 |
| **Phase 3: Reading included studies** | | | |
| *Methods* | | | |
| 9 | Reading and data extraction approach | Describe the reading and data extraction method and processes | Page 11–12 |
| *Results* | | | |
| 10 | Presenting characteristics of included studies | Describe characteristics of the included studies | Page 16–36, Table 5 |
| **Phase 4: Determining how studies are related** | | | |
| *Methods* | | | |
| 11 | Process for determining how studies are related | Describe the methods and processes for determining how the included studies are related:  - Which aspects of studies were compared AND  - How the studies were compared | Page 12 |
| *Methods* |  |  |  |
| 12 | Outcome of relating studies | Describe how studies relate to each other | Page 12 |
| **Phase 5: Translating studies into one another** | | | |
| *Methods* |  |  |  |
| 13 | Process of translating studies | Describe the methods of translation:  - Describe steps taken to preserve the context and meaning of the relationships between concepts within and across studies  -Describe how the reciprocal and refutational translations were conducted  - Describe how potential alternative interpretations or explanations were considered in the translations | Page 12–13, Table 3, Table 4 |
| *Results* |  |  |  |
| 14 | Outcome of translation | Describe the interpretive findings of the translation. | Page 18–50, Table 3 and 4, S3, S4 |
| **Phase 6: Synthesizing translations** | | | |
| *Methods* |  |  |  |
| 15 | Synthesis process | Describe the methods used to develop overarching concepts (“synthesised translations”) Describe how potential alternative interpretations or explanations were considered in the synthesis | Page 17, Table 5, S4, S5 |
| *Results* |  |  |  |
| 16 | Outcome of synthesis process | Describe the new theory, conceptual framework, model, configuration, or interpretation of data developed from the synthesis | Page 18–52 |
| **Phase 7: Expressing the synthesis** | | | |
| *Discussion* |  |  |  |
| 17 | Summary of findings | Summarize the main findings of the translation and synthesis and compare them to existing literature | Page 52–53 |
| 18 | Strengths, limitations, and reflexivity | Reflect on and describe the strengths and limitations of the synthesis:  - Methodological aspects—for example, describe how the synthesis findings were influenced by the nature of the included studies and how the meta-ethnography was conducted.  - Reflexivity | Page 55 |
