# Supplementary material for: Context matters: A meta-ethnography investigating barriers and facilitators for the effective implementation of gambling harm prevention and reduction policies
Source: PLoS One. 2026 Feb 25;21(2):e0343595. doi: 10.1371/journal.pone.0343595 (PMC12935266; doi:10.1371/journal.pone.0343595)
Supplement: S3 File — (DOCX) [file pone.0343595.s003.docx]

**S3 File. Database searches.**

The initial search was conducted in Medline (Ovid), combining subject headings related to gambling, interventions or policies, and qualitative research with equivalent free-text terms in titles, abstracts and author keywords. This strategy was adapted for use in PsycINFO and SocINDEX (both via EBSCO), incorporating database specific search terms (e.g., subject headings for gambling, policy, and qualitative methods). The same core strategy was applied to two ProQuest databases—Worldwide Political Science Abstracts (WPSA) and Applied Social Sciences Index and Abstracts (ASSIA). For the Web of Science Core Collection, a combination of free-text terms and adapted subject terms from previous searches was used. Google Scholar was searched using a modified version of the Web of Science strategy.

| Medline through Ovid  Date 030924 | | |
| --- | --- | --- |
| Nr | Search terms | Items |
| Phenomenon: Gambling behavior | | |
| 1. | Gambling.sh. | 7,243 |
| 2. | (gambling or betting or wager* or casino* or bettor* or gambler* or punter*).ti,ab,kf | 11,176 |
| 3. | 1 or 2 | 11,991 |
| Intervention: harm reduction and prevention policies | | |
| 4. | (social responsibility or government regulation or harm reduction or public health or politics or policy or policy making or public health or public policy or health promotion or government programs or health education).sh. | 374,061 |
| 5. | (policy or policies or intervention or responsible gambling or measure or tool* or regulat* or prevent* or minimi* or reduc* or education* or program* or promotion or strateg* or campaign* or protect*or legisla* or unregulated or offshore or restrict* or limit*).ti,ab,kf. | 13,071,296 |
| 6. | 4 or 5 | 13,234,835 |
| Study design: Qualitative methods | | |
| 7. | (qualitative research or focus groups or grounded theory or hermeneutics).sh | 119,003 |
| 8. | (qualitative or discourse or discourse analysis or thematic analysis or content analysis or framework analysis or narrative or phenomenology* or ethnograph* or interview* or focus group* or document analysis).ti,ab,kf | 861,982 |
| 9. | 7 or 8 | 872,016 |
| Combined sets: | | |
| 10. | 3 and 6 and 9 | 802 |
| Exclusions: | | |
| 11. | (review or meta-analysis or editorial or commentary or protocol*).ti,pt. | 4,605,847 |
| Final | 10 not 11 | 654 |

| APA PsycInfo through EBSCO  Date 03092024 | | |
| --- | --- | --- |
| Nr | Search terms | Items |
| Phenomenon: Gambling behavior | | |
| 1. | MA(gambling or gambling disorder) | 3,956 |
| 2. | TI(gambling or betting or wager* or casino* or bettor* or gambler* or punter*) OR AB(gambling or betting or wager* or casino* or bettor* or gambler* or punter*) OR SU(gambling or betting or wager* or casino* or bettor* or gambler* or punter*) | 15,068 |
| 3. | S1 OR S2 (15 068) | 15,068 |
| Intervention: harm reduction and prevention policies | | |
| 4. | MA(social responsibility or corporate social responsibility or harm reduction or public health or public health research or public health campaigns or politics or policy making or government policy making or health promotion or government programs or intervention or health education or consumer protection) | 44,479 |
| 5. | TI(policy or policies or intervention or responsible gambling or measure* or tool* or regulat* or prevent* or minimi* or reduc* or education* or program* or promotion or strateg* or campaign* or protect*or legisla* or unregulated or offshore or restrict* or limit*) OR AB(policy or policies or intervention or “responsible gambling” or measure* or tool* or regulat* or prevent* or minimi* or reduc* or education* or program* or promotion or strateg* or campaign* or protect*or legisla* or unregulated or offshore or restrict* or limit*) OR SU(policy or policies or intervention or “responsible gambling” or measure* or tool* or regulat* or prevent* or minimi* or reduc* or education* or program* or promotion or strateg* or campaign* or protect*or legisla* or unregulated or offshore or restrict* or limit*) | 3,166,223 |
| 6. | S4 OR S5 | 3,169,923 |
| Study design: Qualitative methods | | |
| 7. | MA(qualitative methods or narrative analysis or interpretative phenomenological analysis or focus groups or grounded theory or hermeneutics or thematic analysis or content analysis or discourse analysis) | 11,263 |
| 8. | TI(qualitative or discourse or discourse analysis or thematic analysis or content analysis or framework analysis or narrative or phenomenology* or ethnograph* or interview* or focus group* or document analysis) OR AB(qualitative or discourse or discourse analysis or thematic analysis or content analysis or framework analysis or narrative or phenomenology* or ethnograph* or interview* or focus group* or document analysis) OR SU(qualitative or discourse or discourse analysis or thematic analysis or content analysis or framework analysis or narrative or phenomenology* or ethnograph* or interview* or focus group* or document analysis) | 741,338 |
| 9. | S7 OR S8 | 741,338 |
| Combined sets: | | |
| 10. | S3 AND S6 AND S9 | 1,270 |
| Exclusions: | | |
| 11. | TI(review or meta-analysis or editorial or commentary or protocol*) OR PZ(review or meta-analysis or editorial or commentary or protocol*) | 279,709 |
| Final | S10 NOT S11 | 1,181 |

| SocIndex through EBSCO  Date 03092024 | | |
| --- | --- | --- |
| Nr | Search terms | Items |
| Phenomenon: Gambling behavior | | |
| 1. | SU(compulsive gambling or gambling behavior or casinos or compulsive gamblers or gambling disorder) | 1,279 |
| 2. | TI(gambling or betting or wager* or casino* or bettor* or gambler* or punter*) OR AB(gambling or betting or wager* or casino* or bettor* or gambler* or punter*) OR TP(gambling or betting or wager* or casino* or bettor* or gambler* or punter*) | 4,898 |
| 3. | S1 OR S2 | 4,999 |
| Intervention: harm reduction and prevention policies | | |
| 4. | SU(social responsibility or government regulation or public health or public health & politics or public health research or policy making or government policy or policy sciences or health promotion or preventive medicine or legislation) | 101,948 |
| 5. | TI(policy or policies or intervention or “responsible gambling” or measure* or tool* or regulat* or prevent* or minimi* or reduc* or education* or program* or promotion or strateg* or campaign* or protect*or legisla* or unregulated or offshore or restrict* or limit*) OR AB(policy or policies or intervention or “responsible gambling” or measure* or tool* or regulat* or prevent* or minimi* or reduc* or education* or program* or promotion or strateg* or campaign* or protect*or legisla* or unregulated or offshore or restrict* or limit*) OR TP(policy or policies or intervention or “responsible gambling” or measure* or tool* or regulat* or prevent* or minimi* or reduc* or education* or program* or promotion or strateg* or campaign* or protect*or legisla* or unregulated or offshore or restrict* or limit*) | 1,212,368 |
| 6. | S4 OR S5 | 1,243,688 |
| Study design: Qualitative methods | | |
| 7. | SU(qualitative research or discourse analysis or critical discourse analysis or focus groups or grounded theory or ethnology or hermeneutics or frames or phenomenology) | 60,326 |
| 8. | TI(qualitative or discourse or discourse analysis or thematic analysis or content analysis or framework analysis or narrative or phenomenology* or ethnograph* or interview* or focus group* or document analysis) OR AB(qualitative or discourse or discourse analysis or thematic analysis or content analysi” or framework analysis or narrative or phenomenology* or ethnograph* or interview* or focus group* or document analysis) OR TP(qualitative or discourse or discourse analysis or thematic analysis or content analysis or framework analysis or narrative or phenomenology* or ethnograph* or interview* or focus group* or document analysis) | 327,847 |
| 9. | S7 OR S8 | 344,050 |
| Combined sets: | | |
| 10. | S3 AND S6 AND S9 | 373 |
| Exclusions: | | |
| 11. | TI(review or meta-analysis or editorial or commentary or protocol*) OR PT(review or meta-analysis or editorial or commentary or protocol*) | 661,956 |
| Final | S10 NOT S11 | 357 |

| Worldwide Political Science Abstracts (ProQuest)  Date 03092024 | | |
| --- | --- | --- |
| Nr | Search terms | Items |
| Phenomenon: Gambling behavior | | |
| 1. | MAINSUBJECT.EXACT(“gambling” or “casinos” or “lotteries” or “slot machines”) | 1,047 |
| 2. | TITLE(gambling or betting or wager* or casino* or bettor* or gambler* or punter*) OR ABSTRACT(gambling or betting or wager* or casino* or bettor* or gambler* or punter*) OR SUBJECT(gambling or betting or wager* or casino* or bettor* or gambler* or punter*) | 1,852 |
| 3. | [S1] OR [S2] | 2,026 |
| Intervention: harm reduction and prevention policies | | |
| 4. | MAINSUBJECT.EXACT(“public policy” or “social responsibility” or “intervention” or “state intervention” or “regulation” or “prevention” or “disease prevention” or “public health education” or “health promotion” or “consumer protection” or “legislation” or “health care policy” or “mental health policy” or “public health”) | 106,331 |
| 5. | . TITLE(policy or policies or intervention or “responsible gambling” or measure* or tool* or regulat* or prevent* or minimi* or reduc* or education* or program* or promotion or strateg* or campaign* or protect*or legisla* or unregulated or offshore or restrict* or limit*) OR ABSTRACT(policy or policies or intervention or “responsible gambling” or measure* or tool* or regulat* or prevent* or minimi* or reduc* or education* or program* or promotion or strateg* or campaign* or protect*or legisla* or unregulated or offshore or restrict* or limit*) OR SUBJECT(policy or policies or intervention or “responsible gambling” or measure* or tool* or regulat* or prevent* or minimi* or reduc* or education* or program* or promotion or strateg* or campaign* or protect*or legisla* or unregulated or offshore or restrict* or limit*) | 728,125 |
| 6. | [S4] OR [S5] | 736,167 |
| Study design: Qualitative methods | | |
| 7. | MAINSUBJECT.EXACT(“qualitative analysis” or “qualitative research” or “qualitative methods” or “qualitative studies” or “discourse analysis” or “content analysis” or “frame analysis” or “phenomenology” or “ethnography” or “interviews” or “focus groups” or “hermeneutics”) | 35,038 |
| 8. | TITLE (qualitative or discourse or “discourse analysis” or “thematic analysis” or “content analysis” or “framework analysis” or narrative or phenomenology* or ethnograph* or interview* or “focus group*” or “document analysis”) OR ABSTRACT(qualitative or discourse or “discourse analysis” or “thematic analysis” or “content analysis” or” framework analysis” or narrative or phenomenology* or ethnograph* or interview* or “focus group*” or “document analysis”) OR SUBJECT(qualitative or discourse or “discourse analysis” or “thematic analysis” or “content analysis” or “framework analysis” or narrative or phenomenology* or ethnograph* or interview* or “focus group*” or “document analysis”) | 167,267 |
| 9. | [S7] OR [S8] | 168,696 |
| Combined sets: | | |
| 10. | [S3] AND [S6] AND [S9] | 150 |
| Exclusions: | | |
| 11. | TITLE(review or meta-analysis or editorial or commentary or protocol*) OR DTYPE(review or meta-analysis or editorial or commentary or protocol*) | 422,560 |
| Final | [S10] NOT [S11] | 143 |

| Applied Social Science Index & Abstracts (ASSIA) through ProQuest  Date  03092024 | | |
| --- | --- | --- |
| Nr | Search terms | Items |
| Phenomenon: Gambling behavior | | |
| 1. | MAINSUBJECT.EXACT(“gambling” or “gambling industry” or “pathological gambling” or “casinos” or “lotteries” or “betting”) | 3,648 |
| 2. | TITLE(gambling or betting or wager* or casino* or bettor* or gambler* or punter*) OR ABSTRACT(gambling or betting or wager* or casino* or bettor* or gambler* or punter*) OR SUBJECT(gambling or betting or wager* or casino* or bettor* or gambler* or punter*) | 4,422 |
| 3. | [S1] OR [S2] | 4,507 |
| Intervention: harm reduction and prevention policies | | |
| 4. | MAINSUBJECT.EXACT(“government policy” or “public policy” or “social policy” or “corporate social responsibility” or “social responsibility” or “intervention” or “state intervention” or “social interventions” or “regulation” or “prevention” or “disease prevention” or “health promotion” or “mental health promotion” or “consumer protection” or “legislation” or “health policy” or “mental health policy” or “public health policy” or “educational programmes” or “preventive programmes” or “harm reduction”) | 108,611 |
| 5. | TITLE(policy or policies or intervention or “responsible gambling” or measure* or tool* or regulat* or prevent* or minimi* or reduc* or education* or program* or promotion or strateg* or campaign* or protect*or legisla* or unregulated or offshore or restrict* or limit*) OR ABSTRACT(policy or policies or intervention or “responsible gambling” or measure* or tool* or regulat* or prevent* or minimi* or reduc* or education* or program* or promotion or strateg* or campaign* or protect*or legisla* or unregulated or offshore or restrict* or limit*) OR SUBJECT(policy or policies or intervention or “responsible gambling” or measure* or tool* or regulat* or prevent* or minimi* or reduc* or education* or program* or promotion or strateg* or campaign* or protect*or legisla* or unregulated or offshore or restrict* or limit*) | 802,159 |
| 6. | [S4] OR [S5] | 805,288 |
| Study design: Qualitative methods | | |
| 7. | MAINSUBJECT.EXACT(“qualitative analysis” or “qualitative research” or “qualitative methods” or “qualitative studies” or “discourse analysis” or “critical discourse analysis” or “content analysis” or “frame analysis” or “phenomenology” or “ethnography” or “interviews” or “focus groups” or “hermeneutics”) | 62,518 |
| 8. | TITLE(qualitative or discourse or “discourse analysis” or “thematic analysis” or “content analysis” or “framework analysis” or narrative or phenomenology* or ethnograph* or interview* or “focus group*” or “document analysis”) OR ABSTRACT(qualitative or discourse or “discourse analysis” or “thematic analysis” or “content analysis” or” framework analysis” or narrative or phenomenology* or ethnograph* or interview* or “focus group*” or “document analysis”) OR SUBJECT(qualitative or discourse or “discourse analysis” or “thematic analysis” or “content analysis” or “framework analysis” or narrative or phenomenology* or ethnograph* or interview* or “focus group*” or “document analysis”) | 207,330 |
| 9. | [S7] OR [S8] | 208,402 |
| Combined sets: | | |
| 10. | [S3] AND [S6] AND [S9] | 419 |
| Exclusions: | | |
| 11. | TITLE(review or meta-analysis or editorial or commentary or protocol*) OR DTYPE(review or meta-analysis or editorial or commentary or protocol*) | 153,560 |
| Final | [S10] NOT [S11] | 386 |

| Web of Science Core Collection  Date 02092024 | | |
| --- | --- | --- |
| Nr | Search terms | Items |
| Phenomenon: Gambling behavior | | |
| 1. | TS=(gambling or betting or wager* or casino* or bettor* or gambler* or punter or “slot machines”) | 114,137 |
| Intervention: harm reduction and prevention policies | | |
| 2. | TS=(politics or policy or policies or intervention or “responsible gambling” or “social responsibility” or “public health” or measure* or tool* or regulat* or prevent* or minimi* or reduc* or education* or program* or promotion or strateg* or campaign* or protect*or legisla* or unregulated or offshore or restrict* or limit*) | 26,566,555 |
| Study design: Qualitative methods | | |
| 3. | TS=(qualitative or discourse or “discourse analysis” or “thematic analysis” or “content analysis” or “framework analysis” or narrative or phenomenology* or ethnograph* or interview* or “focus group*” or “document analysis” or “frame analysis” or “hermeneutic*”) | 1,870,698 |
| Combined sets: | | |
| 4. | #1 AND #2 AND #3 | 2,318 |
| Exclusions: | | |
| 5. | TI=(“systematic review” or “systematic literature review” or “scoping review” or “book review” or “umbrella review” or “meta-analysis” or editorial or commentary or protocol*) | 808,485 |
| Final | #4 NOT #5 | 2,215 |

| Google Scholar (title search) [v4.0]  Date 27082024 | | |
| --- | --- | --- |
| Nr | Search terms | Items |
| Phenomenon: Gambling behavior | | |
| 1. | intitle:gambling\|betting\|casino*\|gambler*\| |  |
| Intervention: harm reduction and prevention policies | | |
| 2. | intitle:politics\|polic*\|intervention*\|responsib*\|public\|regulat*\|prevent*\|minimi*\|  reduc*\|promot*\|protect*\|restrict*\|limit* |  |
| Study design: Qualitative methods | | |
| 3. | intitle:qualitative\|discourse\|thematic\|content\|narrative\|ethnograph*\|interview* |  |
| Combined sets: | | |
| Final | 1 and 2 and 3 in same search | 171 |

| Google Scholar (general search) [v5.0]  Date 28082024 | | |
| --- | --- | --- |
| Nr | Search terms | Items |
| Phenomenon: Gambling behavior | | |
| 1. | gambling\|betting\|casino* |  |
| Intervention: harm reduction and prevention policies | | |
| 2. | politics\|polic*\|intervention*\|responsib*\|public\|regulat*\|prevent*\|minimi*\|reduc*\|  program*\|promot*\|strateg*\|campaign*\|protect*\|legisla*\|restrict*\|limit* |  |
| Study design: Qualitative methods | | |
| 3. | qualitative\|discourse\|thematic\|content\|narrative\|ethnograph*\|interview*\|document |  |
| Combined sets: | | |
| Final | 1 and 2 and 3 in the same search. Results from the first 16 result pages were included. | 160 |
