# Supplementary material for: Context matters: A meta-ethnography investigating barriers and facilitators for the effective implementation of gambling harm prevention and reduction policies
Source: PLoS One. 2026 Feb 25;21(2):e0343595. doi: 10.1371/journal.pone.0343595 (PMC12935266; doi:10.1371/journal.pone.0343595)
Supplement: S4 File — (DOCX) [file pone.0343595.s004.docx]

**S4 File. Synthesis tables.**

Table 1. Educational interventions.

| Methods and themes | Van Schalkwyk et al. (2022) | Torrance et al. (2024) | Van Schalkwyk et al. (2021) | Gainsbury et al. (2018a) | Leung & Kong (2013) | Thomas et al. (2015) | McCarthy et al. (2022) | Newall et al. (2023) | Messerlian & Derevensky (2007) | McCarthy et al. (2023) | Marko et al. (2023) | Pitt et al. (2022) | Van Schalkwyk et al. (2024) |
| --- | --- | --- | --- | --- | --- | --- | --- | --- | --- | --- | --- | --- | --- |
| Sample | Campaign materials | Scholars, experts by experience | Program documents | Young adults, seniors, gamblers | Gamblers of varying risk levels' video testimonials | Gamblers of varying risk levels | Women aged 20-41 | Academics, regulators, treatment providers | Youths aged 12-18 years | Stakeholders, women experts by experience | Gamblers, affected others | Youths aged 11-17 years | Program documents |
| Data collection |  | Focus groups |  | Focus groups |  | Interviews | Interviews | Focus groups | Focus groups | Focus groups | Interviews | Interviews |  |
| Effects of education | Emphasis on learning individual skills to gamble in responsible and rational fashion to resist harmful effects. |  | Campaign presented as contributing to behavior change and being superior to other campaigns. |  |  | Responsible gambling messages produced feelings of shame and stigma in people with problem gambling, less likeliness to seek help, Stigma, and shame major barriers to seek help. | Responsible gambling messages not effective, accurate risk information of products needed. | Responsible gambling messages may prevent treatment seeking. Safer gambling messages not effective for regular gamblers or young men. Tailoring messages by risk level. | Children want warnings on harm. (Responsible gambling messages are supported by some children.) | Instead of underlining personal responsibility, product information and harm information needed. Tailoring messages for women. | Responsible gambling messages considered ineffective by gamblers and affected others. To increase effectiveness of messages, realistic warnings on harm needed. | Responsible gambling messages ineffective and attempt to absolve the industry from responsibility by emphasizing personal responsibility. Realistic warnings on gambling harm. | Presentation of campaigns as effective. |
| The figures of responsible and irresponsible gambler | Rational personal responsibility is offered as a solution to harm. The dichotomy of responsible/rational and irresponsible/irrational gambler/person is produced. |  | Gamblers experiencing harm presented as vulnerable minority lacking control. Dichotomy of gambling as fun for most in control and not fun for those who lack self-control. |  | People with problem gambling affirm their identity through negation by denying their former identity. Social gamblers construct individual identity as rational and self-disciplined. Dichotomy of problem and normal gamblers produced. |  |  |  |  |  |  |  |  |
| The structure and style of messages and videos |  | Educational videos need to be concise and presented in impactful way. Simple and conversational language with psychological content. |  | Young adults appreciated responsive tone instead of accusing tone and simple messages. Older adults appreciated non-accusing tone. Skill Game Gamblers were interested in simple language. |  | Detailed informational content on harms similar to tobacco was supported, and to engage people to dialogue over harm. |  |  |  |  |  |  |  |

Note: refutational evidence in parenthesis.

Table 2. Exclusion programs.

| Methods and themes | Pickering et al. (2022) | Pickering et al. (2019) | Goh et al. (2016) | Hing et al. (2014) | Kraus et al. (2023) |
| --- | --- | --- | --- | --- | --- |
| Sample | Self-excluders, counsellors, venue staff, policy makers | Current and former self-excluders | Family members of people with problem gambling | People with problem gambling (self-excluders and non-excluders) | Gamblers, venue managers, venue staff, |
| Data collection | Focus groups | Interview | Interview | Interview | Interview |
| Barriers to effectiveness |  | Low coverage a challenge. Difficulties in detecting people. | Effectiveness undermined by the possibility to gamble outside casinos. | Availability of alternative venues a challenge. Poor monitoring. Apathy on the part of staff. Not effective. | Possibility of shifting to other gambling venues. Lack of identity checks. Low uptake, not effective. |
| Facilitators and effectiveness | Sharing data with authorities could enhance the supervisions and effectiveness. | Treatment and support considered vital component of self-exclusion by the self-excluders. Longer exclusion times were supported. No revocation. | Family exclusion cause a sense of relief for the close ones. | Possibility to decide the length supported. Effective for some, not personally. | Staff in favor of linking additional support to self-exclusion. Gamblers considered self-exclusion beneficial. Longer exclusion time supported. Some staff supported unlimited length of self-exclusion for people with problems. |
| Registration barriers |  | Registration time-consuming as one had to register to every EGM venue separately. Lack of respect by the staff. Lack of information. |  | Registration in a single venue difficult, and embarrassing. Negative experiences of interactions with the staff. Lack of information on registration. | Staff was reported trying to talk gambler out of the idea of self-excluding. |
| Registration facilitators | The emotional state of the self-excluders needs to be considered. The easiness of use. Ability use smart phone. Validating the decisions. Ability to contact help services. | Compassionate treatment. Possibility to register online may strengthen self-efficacy. Family members or other close ones play a key role. Important that the staff approaches discreetly. |  | Sensitivity and support from staff appreciated. Multi-venue exclusion supported. Support from help services or close ones decisive. | Approaching need to be done with right tone and time. |

Table 3. Spending limits.

| Methods and themes | Gainsbury et al. (2018b Und) | Selin (2022) | Lakew (2022) | Drosatos et al. (2020) | Swanton et al. (2023) |
| --- | --- | --- | --- | --- | --- |
| Sample | EGM gamblers | Company annual reports | Spending limit users | Addiction experts and gamblers | EGM gamblers, 24-76 years of age |
| Data collection | Focus groups | Publicly available data | Interviews | Interviews | Focus groups |
| Effects of spending limits on control, harm, and user experience | People with problem gambling like to have fixed limits to control their spending. Valuable for some. | Calculative human capabilities and self-control endorsed by the limit setting, | Help in controlling gambling behavior, give psychological comfort and decrease spending, including people with problem gambling. For some people with problem gambling the use of budget tool changed gambling for better. | Helpful end effective in limiting spending and maintaining control in online gambling. | Ability to set fixed limits valued. The cashless system with spending limits was considered effective. |
| Facilitators of effectiveness | Extending spending limits across venues would increase value for those in favor. |  | The ability to use the budget tool across websites was appreciated. |  | Bypassing the spending and other limits would be easy without mandatory and universal scheme. |
| Negative effects | Spending limits take enjoyment out of gambling and result in stopping gambling or migrating to other gambling products. Not valuable for some. |  | Negative experiences with the spending limits or their effects rarely mentioned, linked to user experiences, and wishes to improve the budget tool. |  | Not considered personally useful. Migration to other products a consequence. Possibility to overspend when physical currency is not needed. |
| Privacy, stigma, and freedom issues | Who will have access to information? What other gamblers or venue staff would be able to learn about them? Trust: that a system would be helpful to the player and not designed to benefit the government and industry. |  |  |  | Over-regulation and excessive institutional control and monitoring. Concerns over government and gambling industry conflicts of interest. Privacy and security of users’ personal information. Would “[take] away people’s rights,” such as freedom of choice and privacy. |
| Easy and flexible use | Flexible and responsive to gamblers’ preferences easy to access. Simple and easy to use. |  | More freedom to change their budget limit anytime as they see fit. More options for budget setting that include not just a monthly limit but a daily or weekly limit. |  | Willingness to adopt depend on flexibility, such as the ability to set own limits or to toggle settings on or off. |

Table 4. Behavioral interruptions.

| Methods and themes | Hing & Nuske (2012) | Beckett et al. (2020) | Manian et al. (2023) | Landon et al. (2016) | Bowne & Jarldorn (2024) |
| --- | --- | --- | --- | --- | --- |
| Sample | Frontline venue staff | Venue managers and staff | Venue staff | Gamblers of varying risk levels, venue staff | Venue staff |
| Data collection | Interview | Focus group | Interviews | Focus group | Interviews |
| Challenges with interrupting gambling customers at venues | Not knowing the financial or overall situation of the customer. Customers refusal to acknowledge problems. Fear of angry customer responses. The staff: frustration over not being allowed to intervene. | Lack of skills to approach customers with signs harm. Staff: Fear of aggressive response, lack of knowledge of the customers situation. The staff considered the existing legislation and code not allowing to approach customers. | Employees stop approaching if customer is not acknowledging them. Concerns about misidentification of problem gambling behavior without knowing enough of customers situation. Lack of support and guidelines. |  | Lack of training and language. |
| Role conflict | Staff concerned about loss of revenue if customers leave upon approaching. | Fear of sanctions from employer due to customer dissatisfaction and lost revenue. | Challenge of caring for customers welfare and fear of harming the business if approaching customers proactively. This conflict was apparent when serving the VIP customers as interrupting customer´s gambling would result in direct loss of income for the staff. |  | Balancing between profits of the venue and the welfare of the customers, with profit usually prioritized. Fear of direct income loss. |
| Responses to and effectiveness of automated and human interruptions |  | Managers: Identifying signs of gambling harm were considered clear. Procedure regarding assisting and supporting customers who approach staff was clear and working. Senior managers considered the existing practices to be good. |  | Interruptions were considered ineffective by both gamblers and staff. | Staff: automated alarm system used in the EGM ineffective as interruptions do not follow the alarm. Interruptions did not happen even if revenue increased, and overspending was observed. |

Table 5. Advertising restrictions.

| Methods and themes | González Díaz et al. (2024) | Selin (2016) | Pitt et al. (2024) | Pitt et al. (2022) | McCarthy et al. (2022) |
| --- | --- | --- | --- | --- | --- |
| Sample | Stakeholders: government, industry, media, and mutual support groups | Annual reports, documents | Youths aged 12-17 | Youths aged 11-17 | Women aged 20-41 |
| Data collection | Interviews, seminar, press conferences, policy documents and statements | Publicly available data | Focus groups | Interviews | Interviews |
| Regulation and self-regulation | Industry´s regulatory compliance suspected. Government, industry, and media prioritizes self-regulation as regulation bad for business. |  | Broadcasting time limits protecting young people insufficient. Lack of actions and understanding of harm by government. Regulation of social media platforms a solution | Restrictive regulations complex and time-consuming to enforce. Conflict of interest of government makes regulation difficult. |  |
| Interpretation of law | Difficult to interpretate the law (all but government). | Regulator able to establish interpretation of law instead of operator self-regulation. |  |  |  |
| Restrictions and free market | Industry and media oppose restrictions as volume of advertisement will decline naturally due to market consolidation, restrictions a threat to freedom of trade and expression. | Marketing may not create new demand, channeling of the existing natural demand possible. |  |  |  |
| Emotional and behavioral effects of marketing | Annoyance over volume of marketing. |  | Effects on attitudes and decisions of young people. | Individual responsibility reinforced, contradiction in promoting and preventing simultaneously. | Companies target women. |
| Consumer protection and the content of advertisement. | State-owned companies support risk categorization of products and private companies support risk categorization of gamblers. |  | Risk and harm information to counter advertisement and engaging young people in designing counter-messages and in decision-making. | Countering positive content of advertisements with harm information. | Restrictions needed to protect children. |

Table 6. Feedback interventions.

| Methods and themes | Forsström et al. (2017) | Selin (2022) | Swanton et al. (2023) | Landon et al. (2016) | Drosatos et al. (2020) | Forsström et al. (2022) |
| --- | --- | --- | --- | --- | --- | --- |
| Sample | Gamblers | Annual reports | EGM gamblers, aged 24-76 | Gamblers of varying risk levels, venue staff | Addiction experts and gamblers | Gamblers |
| Data collection | Interviews |  | Focus groups | Focus group | Interviews | Questionnaire with an open-ended question |
| Responses, perceived effects, and usefulness | No significant contribution to behavioral change. Few of the users considered the tool to have contributed among other factors to decreased spending. Usually, the tool did not contribute to attitudinal change towards own gambling. | Enhancing or strengthening self-control and responsibility | Helpful in maintaining control and keeping track on spending. Graphical representations of spending recommended. Circumventing if not mandatory. | Some gamblers considered useful. Staff and gamblers did not consider feedback effective. People with problem gambling considered the information irrelevant or ignored. Gamblers and staff found the feedback annoying for gamblers. | Feedback enables change of focus by the gamblers. Visualized feedback was supported, but not in normative way as the others may have different situation. Annoyance over and ignoring of feedback was mentioned as likely response. | Considering the feedback nonsensical. Agreement between the feedback and own understanding of gambling. Helpful for those with gambling problems but not personally. |
| Privacy concerns and Content and accuracy of the feedback | Some considered the tone of feedback easy to read and grasp while others regarded it boring or banal, even accusing. Some held the feedback inaccurate and even insulting. |  | Was considered intrusive. Concerns institutional control and monitoring, negative impact on personal freedom. Concerns about access to sensitive player data. | Accuracy of the given feedback suspected. Privacy concerns related to the content of feedback as it was potentially visible to others at venue. Some staff considered feedback messages as intrusion to people´s leisure activities. |  | Questioning the accuracy or validity of the feedback as there may difference in income or possibly several people using the same account. |

Table 7. Availability regulations.

| Methods and themes | Rolando et al. (2021) | French et al. (2021) | Rolando et al. (2020) | Marionneau & Järvinen-Tassopoulos (2022) |
| --- | --- | --- | --- | --- |
| Sample | Gamblers |  | Stakeholders | Gamblers |
| Data collection | Interviews | Documents, media, ethnographic fieldnotes | Focus groups | Interviews |
| Effects of availability changes | Gamblers consider restrictions effective in reducing EGM gambling, harm and preventing gambling from becoming more severe. Migration to other gambling products. |  | Increased availability led to problems and increased spending. The industry and business argued gamblers with problems migrate to other regions or illegal gambling. | Some able to abstain after the restrictions were removed, including those with problem gambling. Others resumed gambling upon the removal of restrictions, with people with problem gambling starting to gamble heavily again. Restrictions cause migration to other gambling products. |
| Public health and economic justification of availability regulation |  | While justified by public health threat of offshore gambling, clear that financial aims priority, as no public health provisions. | Economic crisis gave impetus for business owners to install EGMs and an incentive to individuals to seek more money via gambling or escape their problems. |  |
