# Supplementary material for: Context matters: A meta-ethnography investigating barriers and facilitators for the effective implementation of gambling harm prevention and reduction policies
Source: PLoS One. 2026 Feb 25;21(2):e0343595. doi: 10.1371/journal.pone.0343595 (PMC12935266; doi:10.1371/journal.pone.0343595)
Supplement: S5 Table — (DOCX) [file pone.0343595.s005.docx]

**S5 Table. Second and third-order interpretations by intervention.**

| Intervention | Theme | Synthesis of second-order interpretations | Third-order interpretations |  |
| --- | --- | --- | --- | --- |
| Educational interventions | Effects | The industry presents education with an emphasis on individual responsibility as effective, while gamblers and other stakeholders consider it ineffective, stigmatizing, and emphasize the need to offer warnings, positive messages, and tailored communication to different population groups and gamblers based on severity. | Industry-produced educational materials, which emphasize individual responsibility as the solution to gambling harm, contradict the demands of other stakeholders for more effective educational interventions. |  |
|  | Responsible gambler | Gambling is presented as a normal product with harm potential, and children and youth are expected to learn to live with this fact by becoming calculative and acting rationally and responsibly. Gamblers experiencing harm are portrayed as a vulnerable minority lacking control. People with problem gambling are constructed as both active and passive, affirming their identity through negation by denying their former identification with local and working-class identity. Social gamblers construct individual identity as rational, global, and self-disciplined. | The dichotomy between the common rational gambler and the uncommon irresponsible gambler in industry and government education highlights that solutions to gambling harm should be found at the individual level. |  |
|  | The structure and style of messages and videos | Educational videos need to be concise to capture the attention of young people, using simple and conversational language. Young adults appreciate a responsive tone, and all participants valued a non-accusatory approach. Many gamblers found the messages less appealing and relevant than the positive and entertaining gambling advertisements. There is a need to engage people in dialogue about harm. | The content and style of the educational messages need to be straightforward and non-accusing. |  |
| Advertising restrictions | Regulation and self-regulation | The government, industry, and media prioritize self-regulation, viewing formal regulation as bad for business and overly complex. Government has a conflict of interest, and its efforts hindered by the government's lack of expertise. | Careful defining of the content and the interpretation of law may be crucial in preventing harm as otherwise industry opposition to regulation and individualized solutions may prevail due to their strong opposition for business interest. |  |
|  | Interpretation of law | There are difficulties in interpreting the law. The regulator was able to establish the interpretation of the law, rather than relying on operator self-regulation; only the channeling of existing demand is possible. |  |  |
|  | Restrictions and free market | The industry and media assume that advertising will naturally decline due to market consolidation, and that restrictions pose a threat to freedom of trade and expression. |  |  |
|  | Perceived emotional and behavioral effects of marketing justify restrictions | Marketing may cause annoyance due to its volume, influence the attitudes and decisions of young people, create the impression that harm is the individual's fault, cause confusion by combining promotion and prevention in the same messages. Tailored products marketed to appeal to women. | Restrictions to marketing messages justified by their impact on attitudes and behavior. |  |
|  | Consumer protection and the content of advertisement. | Providing risk and harm information to counter advertising and engaging young people in designing counter-messages and participating in decision-making. | Inclusion of harm information and engagement of target groups to counter advertisements. |  |
| Availability regulation | Effects | Migration to other gambling products and regions. Restrictions caused relief, reduced gambling, and harm, and prevented harm for gamblers and people with problem gambling. Negative financial consequences for business. Increased availability may lead to increased spending, but after restrictions, some gamblers continue while others may be able to abstain. | Changes in availability are linked to gambling harm, revenue, and substitution behavior (i.e., migration to other gambling products). |  |
|  | Justification of availability policy | Restrictions and liberalization justified by economic and public health arguments by societal and individual actors. | Industry, government, and other stakeholders all use economic justifications for availability policies, which poses a challenge for prevention and public health. |  |
| Exclusion programs | Facilitators of effectiveness | Treatment and support, in addition to self-exclusion, are important. No revocation of self-exclusion, longer durations, and the ability to tailor the length are supported. | Accessibility of exclusion with extensive coverage, as well as support and compassion from staff. |  |
|  | Barriers to effectiveness | Bypassing by gambling in another venue, contradictions between enforcing self-exclusion and generating revenue from self-excluders, and poor monitoring. | Bypassing and lax enforcement of the exclusion hampers effectiveness. |  |
|  | Registration facilitators | Online registration to multiple venues through an easy and safe site may also enhance self-efficacy. Services and close ones play a vital role in the decision to self-exclude. Sensitivity, compassion, and support from staff are appreciated. Providing information makes exclusion more accessible. | Accessibility of exclusion with extensive coverage, as well as support and compassion from staff. |  |
|  | Registration barriers | Laboriousness of registration, lack of respect from staff, and feelings of shame. Lack of information about registration. The conflict between suggesting self-exclusion and the consequent loss of revenue or the risk of staff facing sanctions. | Laboriousness and distressful registration processes and companies prioritizing revenue over actively assisting gamblers. |  |
| Behavioral feedback | Usefulness and effectiveness | Perceived as useful for people experiencing harm, but not for oneself. The effectiveness on harm and behavior is questioned. Some contribution to decreased spending reported. Graphical representations of spending are recommended. Persons with problem gambling considered the information irrelevant or ignored it. Gamblers and staff found the feedback annoying for gamblers. Feedback enables a shift in focus. | Personalized feedback on gambling behavior may enable change of focus, but generally gamblers suspect relevance and find mandatory feedback annoying. |  |
|  | Privacy concerns and content and accuracy of the feedback | The accuracy of the feedback was criticized due to multiple users or varying income levels. The tone of the feedback was easy to read but also regarded as boring, insulting, or intrusive. There were concerns about privacy, data safety, institutional control and monitoring, and the negative impact on personal freedom. Concerns were also raised about access to sensitive player data. | Gamblers often view personalized feedback as intrusive surveillance that threatens their autonomy and may be experienced as accusatory. |  |
| Behavioral interruptions | Barriers to interrupting gambling customers at venues | When customers approach staff, some are confident while others consider the issue private, emotionally laborious, and awkward to discuss. An existing relationship with the customer is helpful. Staff rationalized their inactivity by citing a lack of knowledge about the customer's financial situation and the subjective nature of interpreting signs of harm. Experience and training help in facing customers. There is fear of angry customer responses. Management rationalized inactivity by pointing to the availability of information on support and self-exclusion at the venue. Staff considered the existing legislation and code as not allowing them to approach customers. | Challenges to interrupting risky behavior include the responsibility placed on frontline staff to manage emotionally charged encounters with gamblers experiencing harm. |  |
|  | Role conflict or "walking a tightrope" | Staff were concerned about loss of revenue or potential sanctions if customers left after being approached. This conflict was especially apparent when serving VIP customers at the casino, as interrupting their gambling could result in a direct loss of income for the staff. There was a constant balancing act between the venue's profits and the welfare of the customers, with profit usually being prioritized. Fear of direct income loss was alluded to. | When interrupting risky gambling behavior care for customer welfare, business profits, and employee income often come into direct conflict. |  |
|  | Responses and effectiveness | Identifying gambling harm was clear, and the procedure for assisting customers who approached staff was functioning. Senior managers considered the existing practices to be effective, while floor staff found them insufficient. Automated interruptions were viewed as ineffective by both gamblers and staff. Often, the interruption resulted in no behavioral response. The automated alarm system was also ineffective, as interruptions did not follow the alarm. Interruptions did not occur even when revenue increased, and overspending was observed. | Both automated and human interruptions of risky behavior at EGM venues considered largely ineffective by gamblers and staff but managers considered practices functioning. |  |
| Spending limits | Effects on self-control and harm | People with problem gambling would like to have fixed limits to control their spending. Spending limits are perceived as effective in reducing spending and harm, and in providing psychological comfort, including for people with problem gambling. | Gamblers perceive potentially beneficial effects on spending and self-control. |  |
|  | Factors enhancing effectiveness | Extending spending limits across venues and platforms, and making them mandatory, would increase their effectiveness. | Universal spending limits enhance effectiveness. |  |
|  | Negative effects | Limits were seen as taking the enjoyment out of gambling, potentially leading to stopping gambling or migrating to other gambling products. They were not perceived as personally valuable. Negative experiences were linked to user experiences and a desire to improve the budget tool. Limits were not considered personally useful and were not adopted. | Spending limits may cause negative enjoyment and rejection if not considered relevant to the individual, and migration to other gambling products may also occur. |  |
|  | Privacy, stigma, and freedom issues | There are concerns about endangerment of privacy due to the spending limit scheme, as it is unclear who has access to personal data. The scheme is viewed as over-regulation and excessive monitoring, which limit personal freedom. There is also a possibility that the government or industry could use the data for their own purposes to extract revenue. | Spending limits, tied to personal identification, are seen as tools of government surveillance with potential misuse against players’ interests. |  |
|  | Easy and flexible use | Users and potential users emphasized that changing the limits needs to be flexible. User-friendliness—specifically, ease of use—was also emphasized. | Flexibility and user friendliness in setting the limits. |  |
