# Supplementary material for: Context matters: A meta-ethnography investigating barriers and facilitators for the effective implementation of gambling harm prevention and reduction policies
Source: PLoS One. 2026 Feb 25;21(2):e0343595. doi: 10.1371/journal.pone.0343595 (PMC12935266; doi:10.1371/journal.pone.0343595)
Supplement: S6 File — (DOCX) [file pone.0343595.s006.docx]

**S6 File. Line of argument contributions of the review articles.**

Table 1.

| **Contribution to line of argument** | **The framing, perceived effects, and effectiveness of interventions vary, and the interventions can have unintended consequences.** | | | | | |
| --- | --- | --- | --- | --- | --- | --- |
| Third order interpretations | Industry portrayal individuals as both the cause and solution to gambling harm conflicts with other stakeholders' views. | Marketing restrictions justified by their impact on attitudes and behavior. | Changes in availability are linked to gambling harm, revenue, and substitution behavior. | Interruptions considered ineffective. | Spending limits can aid self-control but may lead to migration. | Feedback may enable change of focus, but relevance suspected. |
| Author, year, ranking (S=satisfactory, K=key) |  |  |  |  |  |  |
| Bowne & Jarldorn (2024) (S) |  |  |  | X |  |  |
| Drosatos et al. (2020) (S) |  |  |  |  | X | X |
| Forsström et al. (2017) (S) |  |  |  |  |  | X |
| Forsström et al. (2022) (S) |  |  |  |  |  | X |
| French et al. (2021) (S) |  |  | X |  |  |  |
| Gainsbury et al. (2018b) (K) |  |  |  |  | X |  |
| González Díaz et al. (2024) (K) |  | X |  |  |  |  |
| Lakew (2022) (S) |  |  |  |  | X |  |
| Landon et al. (2016) (S) |  |  |  | X |  | X |
| Leung & Kong (2013) (K) |  |  |  |  |  |  |
| Manian et al. (2023) (S) |  |  |  | X |  |  |
| Marionneau & Järvinen-Tassopoulos (2022) (S) |  |  | X |  |  |  |
| Marko et al. (2023) (S) | X |  |  |  |  |  |
| McCarthy et al. (2022) (S) | X | X |  |  |  |  |
| McCarthy et al. (2023) (S) | X |  |  |  |  |  |
| Messerlian & Derevensky (2007) (S) | X |  |  |  |  |  |
| Newall et al. (2023) (S) | X |  |  |  |  |  |
| Pitt et al. (2022) (S) | X | X |  |  |  |  |
| Pitt et al. (2024) (K) |  | X |  |  |  |  |
| Rolando et al. (2020) (S) |  |  | X |  |  |  |
| Rolando et al. (2021) (S) |  |  | X |  |  |  |
| Selin (2022) (S) |  |  |  |  | X | X |
| Swanton et al. (2023) (S) |  |  |  |  | X | X |
| Thomas et al. (2015) (K) | X |  |  |  |  |  |
| Torrance et al. (2024) (K) |  |  |  |  |  |  |
| Van Schalkwyk et al. (2021) (K) | X |  |  |  |  |  |
| Van Schalkwyk et al. (2022) (K) | X |  |  |  |  |  |
| Van Schalkwyk et al. (2024) (K) | X |  |  |  |  |  |

Table 2.

| **Contribution to line of argument** | **Addressing gambling harm effectively involves countering the gambling industry's influence and its framing of both the causes and the solutions to gambling harm** | | | | |
| --- | --- | --- | --- | --- | --- |
| Third order interpretations | Careful definition and interpretation of law is crucial, due to industry opposition to regulation. | The dichotomy between the rational gambler and the irresponsible gambler individualizes harm. | The content and style of messages need to be straightforward and non-accusing. | Inclusion of harm information. | Industry, government, and other stakeholders use economic justifications for availability policies. |
| Author, year, ranking (S=satisfactory, K=key) |  |  |  |  |  |
| French et al. (2021) (S) |  |  |  |  | X |
| Gainsbury et al. (2018a) (K) |  |  | X |  |  |
| González Díaz et al. (2024) (K) | X |  |  | X |  |
| Leung & Kong (2013) (K) |  | X |  |  |  |
| McCarthy et al. (2022) (S) |  |  |  | X |  |
| Pitt et al. (2022) (S) | X |  |  | X |  |
| Pitt et al. (2024) (K) | X |  |  | X |  |
| Rolando et al. (2020) (S) |  |  |  |  | X |
| Selin (2016) (S) | X |  |  |  |  |
| Thomas et al. (2015) (K) |  |  | X |  |  |
| Torrance et al. (2024) (K) |  |  | X |  |  |
| Van Schalkwyk et al. (2021) (K) |  | X |  |  |  |
| Van Schalkwyk et al. (2022) (K) |  | X |  |  |  |

Table 3.

| **Contribution to line of argument** | **Lax enforcement and the shifting of responsibility to frontline personnel may result in neglecting the specific needs of individuals affected by gambling harm** | | | | | | | | |
| --- | --- | --- | --- | --- | --- | --- | --- | --- | --- |
| Third order interpretations | Accessible exclusion, support, and compassion from staff. | Laboriousness and distressful registration. | The responsibility placed on frontline staff. | Care for customer welfare and profit in conflict. | Personalized feedback intrusive and threat to autonomy. | Exclusion beneficial, and possibility to tailor the length good. | Bypassing and lax enforcement of exclusion hampers effectiveness. | Spending limits are seen as tools of government surveillance. | Flexibility and user friendliness in setting the limits. |
| Author, year, ranking (S=satisfactory, K=key) |  |  |  |  |  |  |  |  |  |
| Beckett et al. (2020) (S) |  |  | X | X |  |  |  |  |  |
| Bowne & Jarldorn (2024) (S) |  |  | X | X |  |  |  |  |  |
| Forsström et al. (2017) (S) |  |  |  |  | X |  |  |  |  |
| Forsström et al. (2022) (S) |  |  |  |  | X |  |  |  |  |
| Gainsbury et al. (2018b) (K) |  |  |  |  |  |  |  | X | X |
| Goh et al. (2016) (S) |  |  |  |  |  | X | X |  |  |
| Hing & Nuske (2012) (S) |  |  | X | X |  |  |  |  |  |
| Hing et al. (2014) (S) | X | X |  |  |  | X | X |  |  |
| Kraus et al. (2023) (S) | X | X |  |  |  | X | X |  |  |
| Lakew (2022) (S) |  |  |  |  |  |  |  |  | X |
| Landon et al. (2016) (S) |  |  |  |  | X |  |  |  |  |
| Manian et al. (2023) (S) |  |  |  | X |  |  |  |  |  |
| Pickering et al. (2019) (S) | X | X |  |  |  | X | X |  |  |
| Pickering et al. (2022) (K) |  | X |  |  |  |  | X |  |  |
| Swanton et al. (2023) (S) |  |  |  |  | X |  |  | X | X |
